# Fully Reversible Regulation of 8‐17 DNAzyme Activity Using Nucleoside‐Based Diarylethenes

**DOI:** 10.1002/chem.71245

**Published:** 2026-06-08

**Authors:** Jörn Bargstedt, Simon M. Büllmann, Sophia Conwell, Andres Jäschke

**Affiliations:** ^1^ Institute of Pharmacy and Molecular Biotechnology Heidelberg University Heidelberg Germany

**Keywords:** 8–17 DNAzyme, diarylethenes, nucleosides, photochromic DNA, reversible regulation

## Abstract

Controlling enzyme activity with external triggers is a central goal in chemical biology. Light is an especially attractive stimulus because it is noninvasive, offers high spatiotemporal precision, and can reversibly modulate molecular function. In this study, we use nucleoside‐based diarylethenes (DAEs) to achieve light‐dependent control of the RNA‐cleaving deoxyribozyme (DNAzyme) 8–17. Three high‐performing DAE‐modified 2′‐deoxyuridines were incorporated at structurally informed positions within the DNAzyme. A single optimal combination, dU‐Ph*^t^* Bu at position 2.1, afforded efficient, reversible photocontrol: in the photostationary state after ultraviolet irradiation (PSS^UV^), the catalytic rate is reduced ∼11‐fold relative to the all‐open form (OF), while comparison of isolated OF and closed form (CF) reveals an ∼200‐fold difference in activity. The switch is thermally robust and can be cycled repeatedly with minimal loss of function. In contrast, seemingly minor changes in DAE substitution, such as addition of a single methyl group, abolish useful regulation, underscoring the critical interplay between photoswitch structure and biomolecular context.

## Graphical abstract

The site‐specific incorporation of nucleosidic diarylethenes into the 8–17 deoxyribozyme (DNAzyme) identified position 2.1 as optimal. Compound dU‐Ph*^t^* Bu enabled efficient light‐dependent control of catalytic activity, while maintaining thermal stability and reversibility. These results establish nucleosidic diarylethenes as modular tools for the precise optical regulation of biomolecular catalysis.

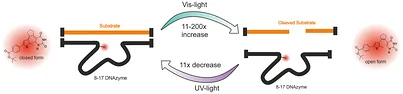

## 1 Introduction

Since their discovery by Breaker and Joyce in 1994 [1]. DNAzymes have emerged as a distinct class of catalytic nucleic acids alongside naturally occurring ribozymes. Motivated by the structural and functional similarities between DNA and RNA, these authors applied an in vitro selection strategy and identified single‐stranded DNA molecules capable of Pb^2^^+^‐dependent cleavage of RNA phosphodiester bonds [1]. In 1997, Santoro and Joyce expanded this concept by generating DNAzymes that cleave a broad range of RNA substrates and, through in vitro selection, isolated the widely studied 8–17 and 10–23 DNAzymes [2]. In parallel, three independent groups identified the 8–17 motif under different experimental conditions [3, 4, 5], demonstrating that its interchangeable binding arms enable recognition of diverse RNA sequences. Owing to this versatility, the 8–17 motif has become a central model system in DNAzyme research [6, 7, 8, 9], frequently used to explore new mechanistic and application‐oriented concepts. A high‐resolution crystal structure of the 8–17 DNAzyme, reported in 2017 [10], complemented earlier extensive studies, including key mutational analyses [8], and provided detailed structural insight into its catalytic mechanism.

Light‐controlled regulation of DNAzyme activity has been pursued to achieve spatiotemporal control over RNA cleavage. The first reported photoregulation of the 8–17 DNAzyme introduced an azobenzene photoswitch as a nucleobase surrogate, yielding an approximately sixfold change in activity upon irradiation [11]. This concept was subsequently extended by appending complementary sequences to the 5' and 3' termini of the DNAzyme and incorporating multiple azobenzene units to modulate hybridization and, consequently, folding of the catalytic core [12]. More recently, a related design was applied to the 10–23 DNAzyme, in which the 3'‐tail was functionalized with multiple azobenzene groups: in the *trans* configuration, intramolecular hybridization between the tail and catalytic domain inhibited substrate binding, whereas in the *cis* configuration, tail dissociation restored DNAzyme activity [13].

Our lab previously developed a variety of nucleoside‐based diarylethenes (DAEs) and incorporated them into synthetic oligonucleotides [14, 15, 16, 17, 18]. Systematic variation of substitution patterns, combined with detailed photochemical and photophysical characterization, yielded robust, reversibly switchable DAE derivatives that efficiently modulate RNA polymerase activity when embedded in promoter sequences [16] and enable the construction of an all‐optical excitonic switch in combination with an acceptor fluorophore [15]. In the present study, top‐performing DAEs from these earlier investigations, together with one newly synthesized derivative, are employed to assess the suitability of nucleosidic DAEs for reversible regulation of 8–17 DNAzyme activity. Different modification sites are evaluated with respect to catalytic performance under UV and visible light irradiation. In the optimized system, the pure open‐ring form (OF) is approximately 200‐fold more active than the pure closed‐ring form (CF). Considering that the UV‐induced photostationary state (PSS^UV^) contains 87% CF and 13% OF, this corresponds to an ∼11‐fold decrease in activity for the PSS mixtures after UV exposure. The switching process is highly reversible upon visible‐light irradiation, with an activity loss of only ∼1.2% per switching cycle, and the DAE‐modified DNAzymes display high thermal and photostability. These findings highlight nucleosidic DAEs as promising tools for light‐controlled regulation of DNAzyme catalysis.

## 2 Results and Discussion

### 2.1 Structural Considerations

**FIGURE 1:**
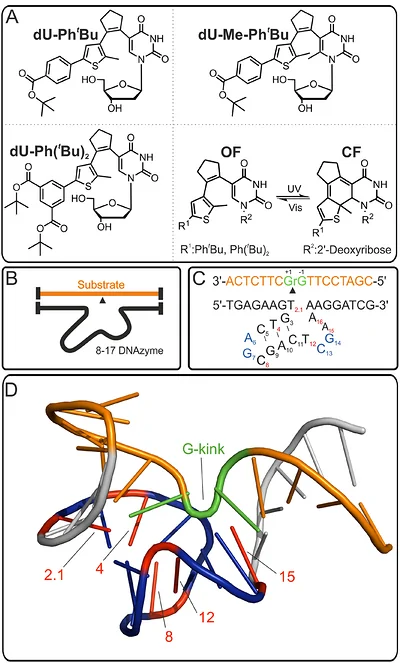
Nucleosidic DAEs used in this work (A), schematic representations (B, C), and 3D‐structure of 8–17 DNAzymes (D) [21, 23]. The sequence of the crystallized DNAzyme differs from that shown in panel C; therefore, residue A_16_ could not be highlighted in panel D.

The development of a photoresponsive deoxyribozyme requires identification of suitable modification sites and an appropriate photoswitch, such that one isomer permits formation of the catalytically competent conformation, whereas the other perturbs it. The CF is more likely to inhibit catalytic activity, as it is sterically more demanding due to the UV‐induced cyclization reaction (Figure 1A). Furthermore, this cyclization alters the orientation of the nucleobase, which may interfere with hydrogen‐bonding interactions (Figure S1). Efficient and reversible photoisomerization is a prerequisite for light‐dependent modulation of catalytic activity. Nucleosidic DAEs are well suited for this purpose, as they integrate seamlessly into DNA structures and, depending on the substitution pattern, can exhibit near‐quantitative switching in both directions. However, the associated changes in structural properties, such as dipole moment and end‐to‐end distance, are subtle relative to other photoswitches [19, 20]. Guided by crystallographic data on 8–17 DNAzymes [21] and previous work on photoresponsive derivatives [22], six positions were selected as promising modification sites (Figure 1): Positions T_2.1_, A_15_, and A_16_ were chosen based on their interactions with the G kink at the substrate cleavage site (Figure S1), while position T_4_ is part of the short stem that stabilizes pairing between G_+1_ – T_2.1_ and G_‐1_ – A_15_. In addition, deletion of C_8_ and T_12_ had previously been shown to cause a pronounced loss of activity, indicating these residues as structurally important and therefore suitable for functional perturbation by modification [10]. The experimental strategy was to first identify the most effective modification position using a highly efficient photoswitch, dU‐Ph*^t^* Bu [16] and subsequently explore more subtle variations in substitution pattern (dU‐Me‐Ph*^t^* Bu [15] and the newly developed dU‐Ph(*^t^* Bu)_2_).

### 2.2 Synthesis and Photochromic Properties of Nucleosidic DAEs

**FIGURE 2:**
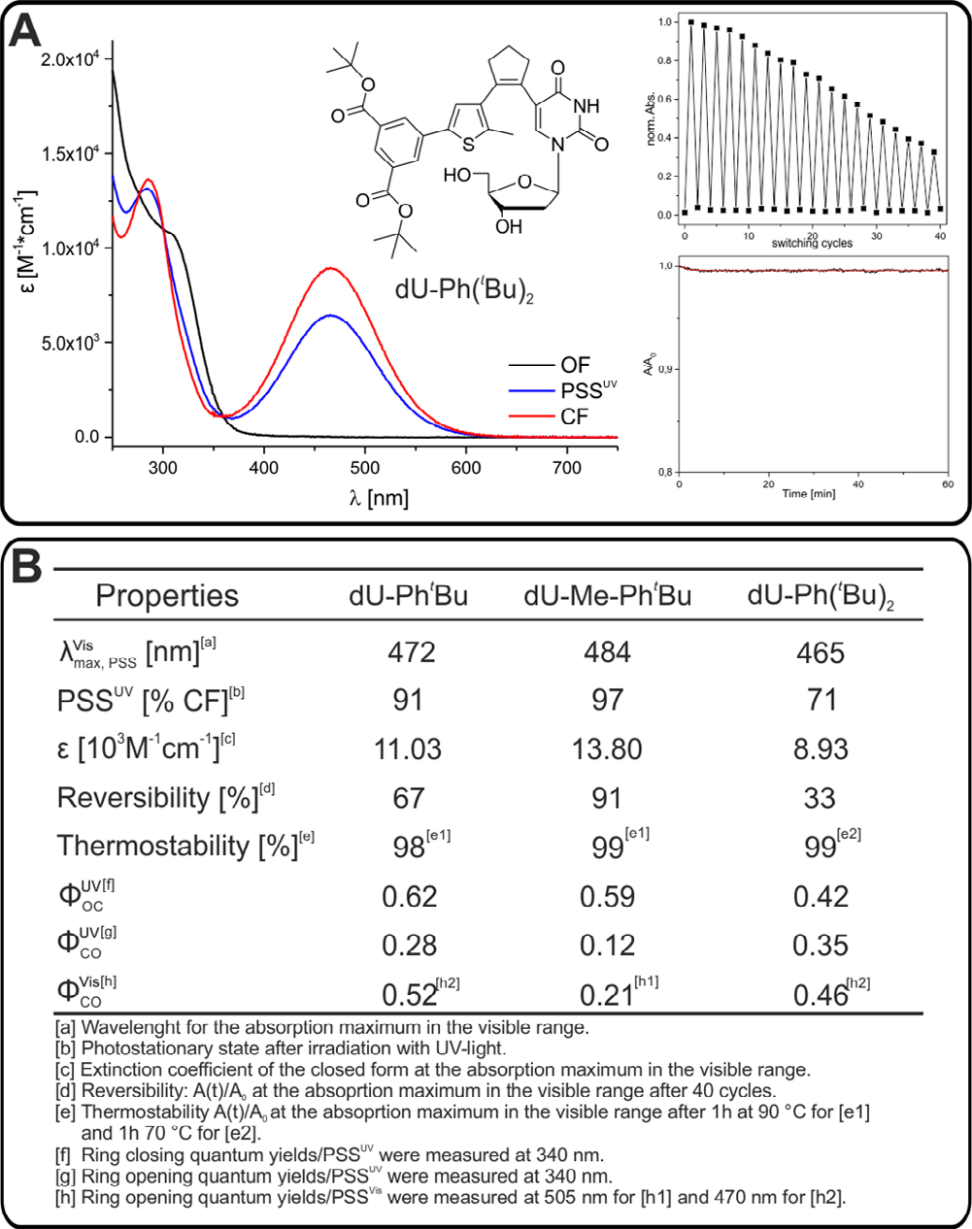
Photophysical properties of nucleosidic DAEs. (A) UV/Vis spectra of dU‐Ph(*^t^* Bu)_2_ OF (black line), PSS^UV^ (blue line) and calculated CF (red line); insets show the reversibility over 40 switching cycles (upper right) and the thermal stability at 70°C (lower right). (B). Comparison of the photophysical properties of dU‐Ph(*^t^* Bu)_2_ with dU‐Ph*^t^* Bu and dU–Me‐Ph*^t^* Bu [15]. DMSO was used as solvent for dU‐Ph*^t^* Bu and dU‐Me‐Ph*^t^* Bu, while dU‐Ph(*^t^* Bu)_2_ was dissolved in water/EtOH (5:1).

DAEs dU‐Ph*^t^* Bu and dU‐Me‐Ph*^t^* Bu were synthesized as previously described [15, 16]. The new DAE dU‐Ph(*^t^* Bu)_2_ was prepared analogously from a thiophene building block and a nucleoside building block, which were subsequently joined by Suzuki cross‐coupling (Scheme S1). The thiophene precursor was obtained from 5‐bromoisophthalic acid via *tert*‐butyl protection of both carboxyl groups to give di‐*tert*‐butyl 5‐bromoisophthalate. Suzuki cross‐coupling with (4‐bromo‐5‐methylthiophen‐2‐yl)boronic acid afforded di‐*tert*‐butyl 5‐(4‐bromo‐5‐methylthiophen‐2‐yl) isophthalate, which was then converted by Miyaura borylation into the corresponding pinacol boronate ester. The nucleoside building block was synthesized from 5‐iodo‐2′‐deoxyuridine: coupling with 2‐(2‐bromocyclopent‐1‐en‐1‐yl)‐4,4,5,5‐tetramethyl‐1,3,2‐dioxaborolane furnished the corresponding boronate intermediate, which was subsequently subjected to Suzuki cross‐coupling with the thiophene precursor to yield dU‐Ph(*^t^* Bu)_2_. As expected, this compound showed marked photochromism, with an intense band around 465 nm appearing upon UV irradiation, complete discoloration after Vis irradiation, and reversible switching behavior (Figure 2A).

Comparison of its photophysical properties with the other two nucleosidic DAEs (Figure 2B) revealed a clear trend. dU‐Me‐Ph*^t^* Bu exhibited the most red‐shifted $\lambda_{max,\;\;PSS}^{Vis}$ (484 nm) and the highest conversion in the PSS^UV^ (97% CF), together with a large extinction coefficient (13.80 × 10^3^ M^−1^ cm^−1^), indicating enhanced conjugation from the methyl substituent. In contrast, dU‐Ph(*^t^* Bu)_2_ showed a blue‐shifted (465 nm), the lowest conversion in the PSS^UV^ (71% CF, Figure S2), and reduced reversibility (33% of the CF initially present in the PSS^UV^ mixture remaining intact after 40 switching cycles, Figure 2A), consistent with steric disruption of planarity. All derivatives were thermally stable with >98% CF initially present in the PSS^UV^ mixture remaining intact after one‐hour incubation at 70 or 90°C. Ring‐opening quantum yields with UV‐light ($\Phi_{CO}^{UV}$) were highest for dU‐Ph(*^t^* Bu)_2_ (0.35), while dU‐Me‐Ph*^t^* Bu displayed the lowest (0.12). Ring opening by visible light was quantitative, as judged from the complete discoloration of the solutions, but ring‐opening yields ($\Phi_{CO}^{Vis}$) were highest for dU‐Ph*^t^* Bu (0.52) and dU‐Ph(*^t^* Bu)_2_ (0.46). Overall, these data show that moderate substitution (Me) optimizes absorption and UV switching, whereas excessive steric bulk impairs reversibility and overall photochemical performance.

### 2.3 Synthesis and Photochromic Properties of DAE‐modified DNAzymes

To render dU‐Ph(tBu)_2_ compatible with solid‐phase synthesis, the previously described synthetic route was modified only at the stage of the nucleoside precursor (Scheme S1). Specifically, 5‐iodo‐2’‐deoxyuridine was first protected at the 5’‐hydroxyl group using a dimethoxytrityl group. All subsequent reactions were carried out as described in the section above. The nucleosidic DAEs were then converted into phosphoramidites, following standard procedures [15, 16], for details see Supplementary Information. The phosphoramidites were incorporated into oligonucleotides by solid‐phase synthesis using *tert*‐butylphenoxyacetyl (tac) protection chemistry (Figure S3). Modified DNA strands were purified using HPLC, analyzed by denaturing 20%‐PAGE and mass spectrometry (Table S1), and subjected to photophysical characterization (Table 1).

**TABLE 1:** Photophysical characterization of the DAE‐modified DNA strands. All measurements were performed in a 30 µM solution in water without the substrate strand. Spectra and chromatograms are shown in Figures S3–S6.

Properties	2.1‐Ph*^t^* Bu	4‐Ph*^t^* Bu	8‐Ph*^t^* Bu	12‐Ph*^t^* Bu	15‐Ph*^t^* Bu	16‐Ph*^t^* Bu	2.1‐Me‐Ph*^t^* Bu	2.1‐Ph(*^t^* Bu)_2_
$\lambda_{max,\;\;PSS}^{Vis}$ [nm]^a^	483	485	476	479	482	481	485	476
PSS^UV^ [% CF]^b^	87	82	77	61	79	83	85	35
ε [10^3^M^−1^cm^−1^]^c^	12.80	15.98	13.92	7.64	16.14	17.37	15.21	10.77
Reversibility [%]^d^	79	89	67	69	93	86	69	59
Thermostabilität [%]^e^	98	97	97	96	98	98	96	93
$\Phi_{OC}^{UV\;}$ ^f^	0.16	0.27	0.13	0.10	0.11	0.11	0.12	0.02
$\Phi_{CO}^{UV}$ ^g^	0.09	0.22	0.04	0.12	0.15	0.09	0.06	0.41
$\Phi_{CO}^{Vis}$ ^h^	0.23	0.24	0.22	0.45	0.16	0.14	0.18	0.27

^a^ Wavelenght of the absorption maximum in the visible range.

^b^ Photostationary state after irradiation with UV‐light.

^c^ Extinction coefficient of the closed form at the absorption maximum in the visible range.

^d^ Reversibility: A(t)/A_0_ at the absorption maximum in the visible range after 40 cycles.

^e^ Thermostability A(t)/A_0_ at the absorption maximum in the visible range after 1 h at 70°C.

^f^ Ring closing quantum yields/PSS^UV^ were measured at 340 nm.

^g^ Ring opening quantum yields/PSS^UV^ were measured at 340 nm.

^h^ Ring opening quantum yields/PSS^Vis^ were measured at 470 nm.

Comparison of the free nucleosides with their DNA‐incorporated counterparts revealed distinct photophysical differences. All DAE‐modified DNAzymes showed a bathochromic shift of 1–13 nm in $\;\lambda_{max,\;\;PSS}^{Vis},$ relative to the free nucleosides, consistent with previous observations for this high‐performing DAE [16]. The new switch dU‐Ph(*^t^* Bu)_2_ followed the same trend, with $\lambda_{max,\;\;PSS}^{Vis}$ shifting from 465 nm (free nucleoside) to 476 nm (incorporated). In all cases, visible‐light irradiation led to complete loss of the visible absorption band and visible discoloration, indicating a PSS^vis^ composition of ∼0% CF.

PSS^UV^ values for the Ph*^t^* Bu‐modified DNAzymes ranged from 61% to 87% CF, compared to 91% CF for the free nucleoside, reflecting reduced ring‐closing ($\phi_{OC}^{UV}$) and the ring‐opening ($\phi_{CO}^{UV}$) quantum yields. The lowest PSS^UV^ values were measured for the 2.1‐Ph(*^t^* Bu)_2_ and 12‐Ph*^t^* Bu variants, where $\phi_{OC}^{UV}$ was lower than $\phi_{CO}^{UV}$. These changes are attributed to altered electronic and steric environments in the DNA context, as solvent effects are known to be minor [24]. For 2.1‐Ph(*^t^* Bu)_2_, PSS^UV^ decreased markedly from 71% CF (free nucleoside) to 35% (incorporated), indicating steric hindrance of CF formation by interactions with the surrounding DNA; similar effects have been observed when either the switch or its environment is sterically demanding (e.g., duplex vs. single‐stranded contexts) [16].

Reversibility measurements over 40 cycles demonstrate the robustness of the incorporated DAE photoswitches (Table 1). As in the free nucleotide (Figure 2B), the new Ph(*^t^* Bu)_2_ switch shows reduced reversibility, compared to the other two DAEs. Thermal stability was high for all six Ph*^t^* Bu‐modified variants. No significant thermally induced ring opening was observed after incubation at 70°C for 1 h, and additional measurements at 25°C and 50°C showed no changes in absorbance. dU‐Ph(*^t^* Bu)_2_ was similarly robust, with 99% of the free nucleoside remaining in the PSS^UV^ and 93% CF retained for the DNA‐incorporated switch, confirming its suitability for applications requiring elevated temperatures and repeated switching.

### 2.4 Determination of DNAzyme Activity as a Function of Irradiation

**FIGURE 3:**
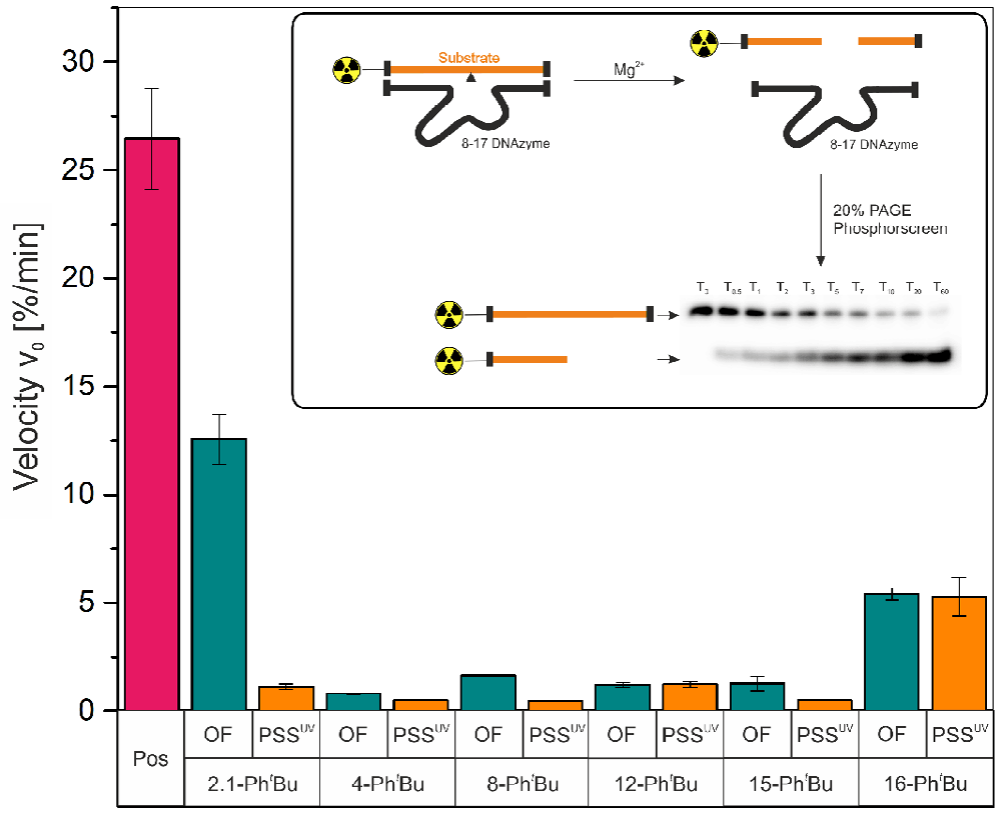
Measured velocities of the unmodified and modified 8–17 DNAzymes. The red bar represents the velocity of the unmodified 8–17 DNAzyme. The inset illustrates the schematic workflow for determining velocities. T_x_ denotes the aliquot collected at t = x min. For gel images and quantification, see Figure S8. The error bars represent the standard deviation of three replicates (n  =  3).

To assess the effect of photoswitching on DNAzyme activity, several assay formats were evaluated. Fluorescence‐based assays were excluded due to potential interference between the excitation light and the wavelengths required for photoswitching. Instead, a classical gel‐based assay using a 5’‐^32^P‐labeled substrate strand was employed. Substrate cleavage was monitored under single‐turnover conditions (4.5‐fold excess of DNAzyme over substrate) either before irradiation (OF) or after UV irradiation to generate the PSS^UV^. Aliquots were taken at defined time points and analyzed by denaturing PAGE; from the intensity ratio of cleaved versus uncleaved bands, percent conversion and reaction rates were determined (Figure 3).

**FIGURE 4:**
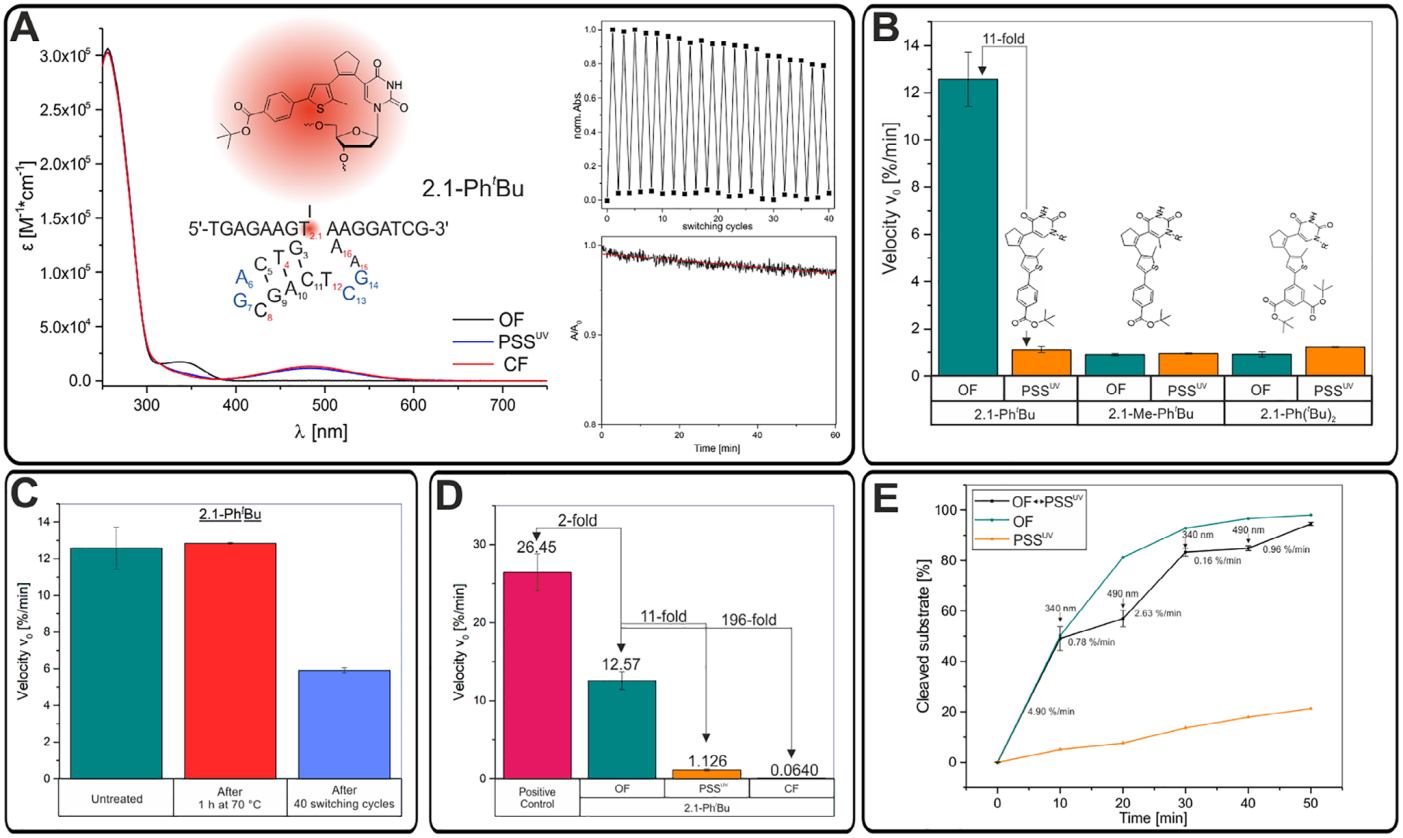
Properties of the 2.1‐Ph^*t*^Bu‐modified DNAzyme (A) UV‐Vis spectrum, reversibility, and thermal stability of the 2.1‐Ph*^t^* Bu catalyst strand. (B) Rates of substrate strand cleavage for DNAzymes containing different DAEs at position 2.1. (C) Rates of substrate strand cleavage for the 2.1‐Ph^*t*^Bu‐modified DNAzyme following thermal stability and reversibility treatment (alternating 340 nm/490 nm irradiation). (D) Switching factors for 2.1‐Ph*^t^* Bu states. The unmodified 8–17 DNAzyme served as the positive control. (E) Reversible switching of DNAzyme activity by alternating 340 nm/490 nm irradiation (black line) and v_0_ values of each step. The pure OF (green) and the PSS^UV^ mixture (orange) were used as controls. For gel images and quantification, see Figures S9–S12. In B‐E, the error bars represent the standard deviation of three replicates (n  =  3).

These data show that the Ph*^t^* Bu modification at position 2.1 enables an 11‐fold change in activity upon light‐induced switching, whereas modifications at positions 4, 8, 12, 15, and 16 reduced DNAzyme activity in both the OF and PSS^UV^ states. Among these, the position‐16 variant retained the highest residual activity in the OF, but its activity in the PSS^UV^ mixture was essentially unchanged, and thus did not exhibit switchable behavior. As only the 2.1‐modified DNAzyme displayed clear light‐dependent regulation, this variant was investigated in more detail (Figure 4A). Photophysical characterization indicated a PSS^UV^ composition of 87% CF and 13% OF. Attempts to further increase the CF fraction in PSS^UV^ by varying irradiation conditions were unsuccessful, prompting the introduction of dU‐Me‐Ph*^t^* Bu and dU‐Ph(*^t^* Bu)_2_at position 2.1.

In the first variation, the dU‐Me‐Ph*^t^* Bu switch was introduced [15]. The additional methyl group at the 6‐position had previously been shown to enhance photochromic performance by increasing resistance to oxidation; however, incorporation of this DAE caused a pronounced decrease in DNAzyme activity in both the OF and PSS^UV^ states (Figure 4B).

The newly developed dU‐Ph(*^t^* Bu)_2_ switch was likewise installed at position 2.1, but this modification also led to a strong reduction in activity in both isomeric forms, with activities lower than those observed for the PSS^UV^ of the dU‐Ph*^t^* Bu‐modified DNAzyme. These findings identify dU‐Ph*^t^* Bu at position 2.1 as the only DAE variant in this series that provides substantial light‐dependent regulation while maintaining sufficient catalytic activity.

### 2.5 Properties of the 2.1‐Ph*^t^* Bu‐modified DNAzyme

To assess the robustness of the 2.1‐Ph*^t^* Bu‐modified DNAzyme, residual catalytic activity was measured after exposure to the conditions used for thermal and photostability testing, namely incubation for 1 h at 70°C or 40 continuous photoswitching cycles (Figure 4C). After thermal treatment, the reaction velocity was 12.8%/min, essentially identical to that of the untreated sample (12.5 %/min), consistent with the observed 99% photochemical stability over 1 h and indicating that the DNAzyme remains structurally and functionally intact under these conditions. In contrast, after 40 switching cycles, the velocity decreased to 5.9 %/min, approximately half of the initial value. This reduction cannot be fully rationalized by the measured reversibility of 79%; if the lost 21% corresponded to catalytically inactive DNAzyme, an activity of 9.9 %/min would be expected. The additional loss is therefore likely due to DNA photodamage, most plausibly arising from photochemical side reactions such as thymidine dimer formation under prolonged UV irradiation (Figure S14) [25, 26, 27, 28, 29]. Thus, incomplete reversibility combined with UV‐induced degradation accounts for the observed decrease in activity, yet an average loss of only ∼1.2% per switching cycle still reflects high overall robustness.

Comparison of the catalytic velocities of the OF and PSS^UV^ states revealed an 11‐fold difference (Figure 4D). Because the PSS^UV^ contains 13% OF, this may largely account for the residual activity in this state. HPLC‐isolated CF exhibited a reaction velocity of only 0.064%/min, corresponding to an approximately 200‐fold difference relative to the OF, placing this system among the most strongly light‐regulated catalysts reported to date [30, 31]. The CF proved to be stable upon storage at ‐21°C in the dark, with no detectable reformation of OF after one month (Figure S15).

### 2.6 Reversible Switching of DNAzyme Activity

To demonstrate reversible control of DNAzyme catalysis, we designed an experiment in which the DAE was repeatedly switched between its OF and CF using alternating irradiation at 340 nm and 490 nm [13]. Prior to each irradiation step, samples were heated to 70°C for 3 min to denature secondary structures, after which an aliquot was taken for PAGE analysis and the remaining sample irradiated as outlined in Figure 4E.

The two control conditions confirmed the previously observed ∼11‐fold difference in activity between OF and PSS^UV^. For the 2.1‐Ph*^t^* Bu‐modified DNAzyme, a staircase‐like progression of substrate cleavage was observed (black trace). During the initial 10 min in the OF, ∼50% of the substrate was cleaved. Subsequent irradiation at 340 nm generated the PSS^UV^, leading to a strong reduction in activity and only a modest further increase in cleavage to ∼57%. Irradiation at 490 nm then restored the OF, resulting in ∼83% cleavage after another 10 min. A second PSS^UV^ period produced only ∼1% additional cleavage, whereas the final OF phase yielded a further ∼10% increase. These data clearly demonstrate that 8‐17 DNAzyme activity can be reversibly and repeatedly modulated by light.

### 2.7 Substrate Dependence of DNAzyme Photomodulation

**FIGURE 5:**
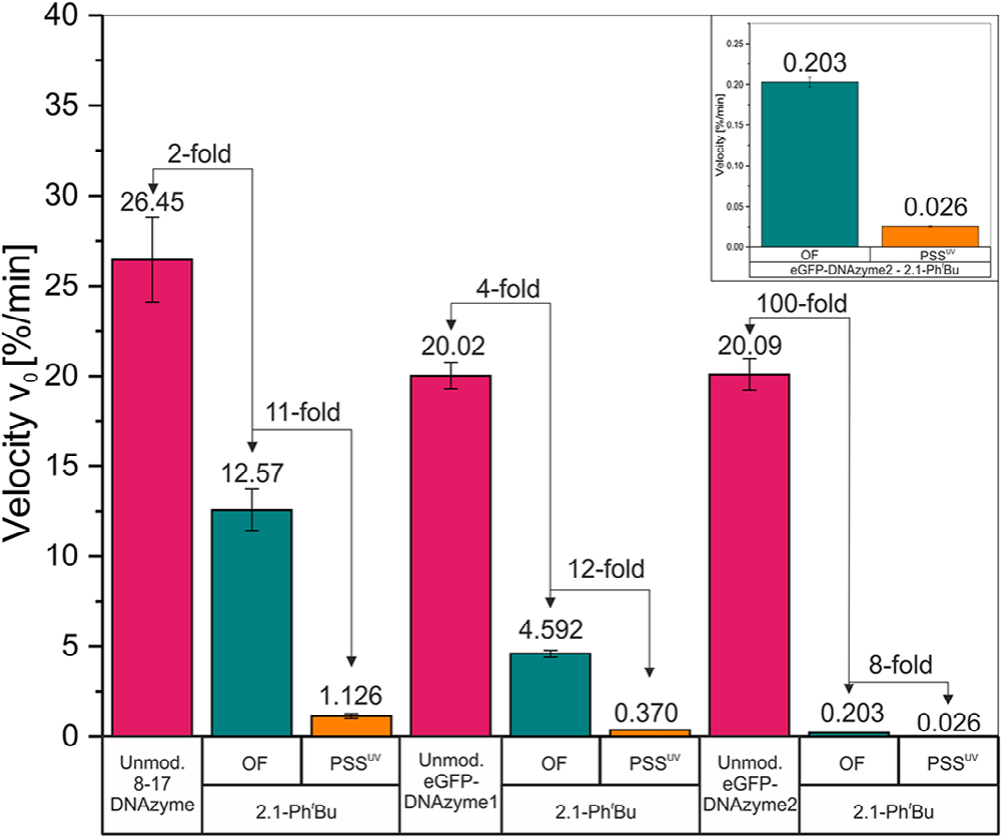
Switching factors for each DNAzyme modified with the dU‐Ph*^t^* Bu at the 2.1 position. Indicated is the x‐fold change from the unmodified DNAzyme (red bar) toward the modified DNAzyme in its OF (green bar) and from the OF toward the PSS^UV^ (orange bar). The inset in the upper right corner shows a zoom‐in of the OF and PSS^UV^ values of the 2.1‐modified eGFP‐DNAzyme2. For gel images and quantification, see Figure S13. The error bars represent the standard deviation of three replicates (n  =  3).

To test the general applicability of our approach, the substrate strand was varied while maintaining a constant catalytic core, including the 2.1‐Ph^*t*^Bu modification (Table S1). Two versions of the modified 8–17 DNAzyme were designed to target different regions of eGFP mRNA. The first construct (eGFP‐DNAzyme1) was adapted from Zhou et al. [32] and targets nucleotides 207–224, whereas the second construct (eGFP‐DNAzyme2) was designed in this study to target nucleotides 277–294. Both unmodified constructs exhibited comparable catalytic performance (Figure 5).

Upon incorporation of dU‐Ph*^t^* Bu at the 2.1 position, eGFP‐DNAzyme1 displayed a fourfold reduction in activity relative to its unmodified counterpart. This decrease was even more pronounced for eGFP‐DNAzyme2, where the open form (OF) of the modified construct exhibited an approximately 100‐fold reduction in activity compared to the unmodified DNAzyme. In contrast, when comparing the OF v_0_ and PPS^UV^ v_0_, the differences were less pronounced. The first construct showed a 12‐fold change, comparable to that of the initial DNAzyme construct (11‐fold), whereas the second construct exhibited a slightly lower switching efficiency, with an approximately eight‐fold change.

These results indicate that incorporation of dU‐Ph*^t^* Bu at the 2.1 position consistently induces a significant, photoresponsive modulation of catalytic activity, largely independent of the substrate. However, the initial reduction in activity resulting from the introduction of dU‐Ph*^t^* Bu strongly depends on the choice of the target region. A comparison of the sequences outside the catalytic core suggests that the nucleotide immediately 5′ to position 2.1 may play an important role. In both the initial 8–17 DNAzyme and eGFP‐DNAzyme1, this position is occupied by a G, whereas in eGFP‐DNAzyme2 it is a T, correlating with substantially reduced residual activity. In the literature, many engineered 8–17 DNAzymes feature either G or C at this position [33, 34, 35, 36]. Furthermore, Liu et al. [10] reported that a G‐C pair at this position stabilizes the structure via base stacking interactions. Nevertheless, this observation requires further structural investigation of the modified construct to be conclusively validated.

## 3 Conclusion

In this study, we integrated several high‐performance nucleosidic diarylethenes into the 8‐17 DNAzyme and identified a single, particularly effective modification site at position 2.1. Incorporation of dU‐Ph*^t^* Bu at this position yielded an ∼11‐fold light‐dependent change in activity in the PSS^UV^ mixture, representing the most effective single modification in an RNA‐cleaving DNAzyme reported to date [37]. Isolation of the pure CF from the PSS^UV^ eliminated residual activity from the remaining 13% OF and revealed an ∼200‐fold difference in reaction velocity between OF and CF. The results in Figure 5 indicate that the difference in v_0_ between OF und PSS^UV^ state is maintained with dU‐Ph*^t^* Bu at the 2.1 position despite variation of the substrate. By contrast, more strongly substituted switches that were promising at the nucleoside level (dU‐Me‐Ph*^t^* Bu and dU‐Ph(*^t^* Bu)_2_) substantially impaired catalytic activity when incorporated at the same position, despite their favorable photophysical properties. Nevertheless it can not be excluded that dU‐Me‐Ph*^t^* Bu and dU‐Ph(*^t^* Bu)_2_ may exert a more pronounced effect at positions other than 2.1 or when targeting a different substrate.

These results underscore that, beyond identifying a responsive site within the biomolecular scaffold, the precise choice of photoswitch is critical; even a single additional methyl group can abolish useful regulation. For the optimal combination (dU‐Ph*^t^* Bu at position 2.1), the resulting DNAzyme is robust, thermally stable up to 70°C, enabling reliable control of activity under harsh conditions. The system can be reversibly switched over many irradiation cycles, with only ∼1.2% activity loss per cycle, making it potentially suitable for applications in photopharmacology, or as light‐activated precision tools in molecular or synthetic biology. The isolated CF is essentially inactive yet chemically and photochemically stable at ‐21°C in the dark and can be activated exclusively by light. Together, these features highlight nucleosidic DAEs, particularly dU‐Ph*^t^* Bu, as powerful tools for the precise, reversible optical control of DNAzyme catalysis. This approach further establishes a modular design principle that can be extended to DNAzymes with tailored binding arms targeting diverse RNA substrates for therapeutic and biotechnological applications, such as gene silencing [32]. Other conceivable applications involve repetitive sensing or logic operations in nucleic acid‐based devices, where the same DNAzyme construct must be switched many times without loss of function (e.g., for cyclic monitoring of metabolites or ions in microfluidic or in vivo‐like environments) [38], where photoswitchable DNAzymes may offer advantages over other, less reversible or less photostable regulation strategies, such as photocleavable caging groups or irreversible strand‐displacement schemes.

## Conflicts of Interest

The authors declare no conflicts of interest.

## Supporting information



Additional supporting information can be found online in the Supporting Information section. The authors have cited additional references within the Supporting Information [39–41].
**Supporting file**: chem71245‐sup‐0001‐SuppMat.pdf.

## Data Availability

The data that supports the findings of this study are available in the supplementary material of this article.

## References

[1] R. R. Breaker and G. F. Joyce , “A DNA Enzyme That Cleaves RNA,” Chemistry & Biology 1 (1994): 223–229. 10.1016/1074-5521(94)90014-0 9383394

[2] S. W. Santoro and G. F. Joyce , “A General Purpose RNA‐cleaving DNA Enzyme,” Proceedings of the National Academy of Sciences 94 (1997): 4262–4266. 10.1073/pnas.94.9.4262 PMC20710 9113977

[3] D. Faulhammer and M. Famulok , “Characterization and Divalent Metal‐ion Dependence of in Vitro Selected Deoxyribozymes Which Cleave DNA/RNA Chimeric Oligonucleotides,” Journal of Molecular Biology 269 (1997): 188–202. 10.1006/jmbi.1997.1036 9191064

[4] R. P. Cruz , J. B. Withers , and Y. Li , “Dinucleotide Junction Cleavage Versatility of 8–17 Deoxyribozyme,” Chemistry & Biology 11 (2004): 57–67. 10.1016/j.chembiol.2003.12.012 15112995

[5] J. Li , W. Zheng , A. H. Kwon , and Y. Lu , “In Vitro Selection and Characterization of a Highly Efficient Zn (II)‐dependent RNA‐cleaving Deoxyribozyme,” Nucleic Acids Research 28 (2000): 481–488. 10.1093/nar/28.2.481 PMC102519 10606646

[6] J. Liu and Y. Lu , “Improving Fluorescent DNAzyme Biosensors by Combining Inter‐and Intramolecular Quenchers,” Analytical Chemistry 75 (2003): 6666–6672. 10.1021/ac034924r 14640743

[7] S. Chakraborti and A. C. Banerjea , “Inhibition of HIV‐1 Gene Expression by Novel DNA Enzymes Targeted to Cleave HIV‐1 TAR RNA: Potential Effectiveness Against all HIV‐1 Isolates,” Molecular Therapy 7 (2003): 817–826. 10.1016/s1525-0016(03)00096-0 12788656

[8] A. Peracchi , M. Bonaccio , and M. Clerici , “A Mutational Analysis of the 8–17 Deoxyribozyme Core,” Journal of Molecular Biology 352 (2005): 783–794. 10.1016/j.jmb.2005.07.059 16125199

[9] A. Vlassov , H. Ilves , and B. Johnston , “Inhibition of hepatitis C IRES‐mediated Gene Expression by 8–17 Deoxyribozymes in human Tissue Culture Cells,” Doklady Biochemistry and Biophysics 410 (2006): 257–259. 10.1134/s1607672906050012 17286096

[10] H. Liu , X. Yu , Y. Chen , et al., “Crystal Structure of an RNA‐cleaving DNAzyme,” Nature Communications 8 (2017): 2006. 10.1038/s41467-017-02203-x PMC5722873 29222499

[11] Y. Liu and D. Sen , “Light‐Regulated Catalysis by an RNA‐cleaving Deoxyribozyme,” Journal of Molecular Biology 341 (2004): 887–892. 10.1016/j.jmb.2004.06.060 15328600

[12] M. Zhou , X. Liang , T. Mochizuki , and H. Asanuma , “A Light‐Driven DNA Nanomachine for the Efficient Photoswitching of RNA Digestion,” Angewandte Chemie International Edition 49 (2010): 2167–2170. 10.1002/anie.200907082 20175178

[13] Y. Kamiya , Y. Arimura , H. Ooi , K. Kato , X.‐G. Liang , and H. Asanuma , “Development of Visible‐Light‐Responsive RNA Scissors Based on a 10–23 DNAzyme,” Chembiochem 19 (2018): 1305–1311. 10.1002/cbic.201800020 29682882

[14] C. Sarter , S. Dey , and A. Jäschke , “Photoswitchable Oligonucleotides Containing Different Diarylethene‐Modified Nucleotides,” ACS Omega 4 (2019): 12125–12129. 10.1021/acsomega.9b01070 PMC6682051 31460326

[15] S. M. Büllmann , T. Kolmar , N. F. Zorn , J. Zaumseil , and A. Jäschke , “A DNA‐Based Two‐Component Excitonic Switch Utilizing High‐Performance Diarylethenes,” Angewandte Chemie International Edition 61 (2022): e202117735. 10.1002/anie.202117735 PMC9305942 35076154

[16] T. Kolmar , S. M. Büllmann , C. Sarter , K. Höfer , and A. Jäschke , “Development of High‐Performance Pyrimidine Nucleoside and Oligonucleotide Diarylethene Photoswitches,” Angewandte Chemie International Edition 60 (2021): 8164–8173. 10.1002/anie.202014878 PMC8049081 33476096

[17] T. Kolmar , A. Becker , R. A. Pfretzschner , A. Lelke , and A. Jäschke , “Development of Red‐Shifted and Fluorogenic Nucleoside and Oligonucleotide Diarylethene Photoswitches,” Chemistry–A European Journal 27 (2021): 17386–17394. 10.1002/chem.202103133 PMC9298058 34519390

[18] S. M. Büllmann , T. Kolmar , P. Slawetzki , S. Wald , and A. Jäschke , “Optochemical Control of Transcription by the Use of 7‐deaza‐adenosine‐based Diarylethenes,” Chemical Communications 57 (2021): 6596–6599. 10.1039/d1cc02639a 34114572

[19] J. Bargstedt , M. Reinschmidt , L. Tydecks , T. Kolmar , C. M. Hendrich , and A. Jäschke , “Photochromic Nucleosides and Oligonucleotides,” Angewandte Chemie International Edition 63 (2024): e202310797. 10.1002/anie.202310797 37966433

[20] M. Marcon , C. Haag , and B. König , “Photoswitches Beyond Azobenzene: A Beginner's Guide,” Beilstein Journal of Organic Chemistry 21 (2025): 1808–1853. 10.3762/bjoc.21.143 PMC12434931 40959513

[21] J. Wieruszewska , A. Pawłowicz , E. Połomska , K. Pasternak , Z. Gdaniec , and W. Andrałojć , “The 8–17 DNAzyme Can Operate in a Single Active Structure Regardless of Metal Ion Cofactor,” Nature Communications 15 (2024): 4218. 10.1038/s41467-024-48638-x PMC11101458 38760331

[22] Y. Watanabe and K. Fujimoto , “Complete Photochemical Regulation of 8–17 DNAzyme Activity by Using Reversible DNA Photo‐crosslinking,” Chembiochem 21 (2020): 3244–3248. 10.1002/cbic.202000227 32596920

[23] A. Peracchi , “Preferential Activation of the 8–17 Deoxyribozyme by Ca 2+ Ions: Evidence for the Identity of 8–17 With the Catalytic Domain of the Mg5 Deoxyribozyme,” Journal of Biological Chemistry 275 (2000): 11693–11697. 10.1074/jbc.275.16.11693 10766789

[24] Y. Ishibashi , T. Umesato , M. Fujiwara , et al., “Solvent Polarity Dependence of Photochromic Reactions of a Diarylethene Derivative as Revealed by Steady‐State and Transient Spectroscopies,” The Journal of Physical Chemistry C 120 (2016): 1170–1177.

[25] C. Crean , Y. Uvaydov , N. E. Geacintov , and V. Shafirovich , “Oxidation of Single‐Stranded Oligonucleotides by Carbonate Radical Anions: Generating Intrastrand Cross‐Links Between Guanine and Thymine Bases Separated by Cytosines,” Nucleic Acids Research 36 (2008): 742–755. 10.1093/nar/gkm1092 PMC2241916 18084033

[26] J. T. Sczepanski , A. C. Jacobs , A. Majumdar , and M. M. Greenberg , “Scope and Mechanism of Interstrand Cross‐Link Formation by the C4′‐oxidized Abasic Site,” Journal of the American Chemical Society 131 (2009): 11132–11139. 10.1021/ja903404v PMC2745085 19722676

[27] J. Cadet , J.‐L. Ravanat , M. TavernaPorro , H. Menoni , and D. Angelov , “Oxidatively Generated Complex DNA Damage: Tandem and Clustered Lesions,” Cancer Letters 327 (2012): 5–15. 10.1016/j.canlet.2012.04.005 22542631

[28] S. Silerme , L. Bobyk , M. Taverna‐Porro , C. Cuier , C. Saint‐Pierre , and J.‐L. Ravanat , “DNA‐Polyamine Cross‐Links Generated Upon One Electron Oxidation of DNA,” Chemical Research in Toxicology 27 (2014): 1011–1018. 10.1021/tx500063d 24798911

[29] M. Kciuk , B. Marciniak , M. Mojzych , and R. Kontek , “Focus on UV‐induced DNA Damage and Repair—Disease Relevance and Protective Strategies,” International Journal of Molecular Sciences 21 (2020): 7264. 10.3390/ijms21197264 PMC7582305 33019598

[30] R. S. Stoll , M. V. Peters , A. Kuhn , et al., “Photoswitchable Catalysts: Correlating Structure and Conformational Dynamics With Reactivity by a Combined Experimental and Computational Approach,” Journal of the American Chemical Society 131 (2009): 357–367. 10.1021/ja807694s 19061327

[31] R. Dorel and B. L. Feringa , “Photoswitchable Catalysis Based on the Isomerisation of Double Bonds,” Chemical Communications 55 (2019): 6477–6486. 10.1039/c9cc01891c 31099809

[32] Z. Zhou , W. Sun , and Z. Huang , “DNAzyme Silencing Gene Expression in Cells via Cleavage and Antisense,” Molecules 28 (2022): 286. 10.3390/molecules28010286 PMC9821912 36615479

[33] M. Cepeda‐Plaza , C. E. McGhee , and Y. Lu , “Evidence of a General Acid–Base Catalysis Mechanism in the 8–17 DNAzyme,” Biochemistry 57 (2018): 1517–1522. 10.1021/acs.biochem.7b01096 PMC5879137 29389111

[34] P. Sang , G. Lu , D. Yu , et al., “Simultaneous Determination of Antibiotics, Mycotoxins, and Hormones in Milk by an 8–17 DNAzyme‐Based Enzyme‐Linked Immunosorbent Assay,” Journal of Agricultural and Food Chemistry 70 (2022): 12681–12688. 10.1021/acs.jafc.2c03833 36037443

[35] D. Mazumdar , N. Nagraj , H.‐K. Kim , et al., “Activity, Folding and Z‐DNA Formation of the 8–17 DNAzyme in the Presence of Monovalent Ions,” Journal of the American Chemical Society 131 (2009): 5506–5515. 10.1021/ja8082939 PMC2765493 19326878

[36] W. Wang , G. Li , X. Zhou , et al., “Investigation of the DNA Methylation‐Modified 8–17 DNAzyme Functions via Sensitive Catalytic Hairpin Self‐Assembly Reaction,” ACS Sensors 10 (2025): 4371–4382. 10.1021/acssensors.5c00581 40387144

[37] S. Keiper and J. S. Vyle , “Reversible Photocontrol of Deoxyribozyme‐Catalyzed RNA Cleavage Under Multiple‐Turnover Conditions,” Angewandte Chemie International Edition 45 (2006): 3306–3309. 10.1002/anie.200600164 16619331

[38] E. M. McConnell , I. Cozma , Q. Mou , J. D. Brennan , Y. Lu , and Y. Li , “Biosensing With Dnazymes,” Chemical Society Reviews 50 (2021): 8954–8994. 10.1039/d1cs00240f PMC9136875 34227631

