# Supplementary material for: Fully Reversible Regulation of 8‐17 DNAzyme Activity Using Nucleoside‐Based Diarylethenes
Source: Chemistry. 2026 Jun 8;32(35):e71245. doi: 10.1002/chem.71245 (PMC13587643; doi:10.1002/chem.71245)
Supplement: Supplementary file 1 [file CHEM-32-e71245-s001.pdf]

Chemistry-A European Journal

Supporting Information

Fully reversible regulation of 8-17 DNAzyme activity using nucleoside-based diarylethenes

Jörn Bargstedt, Simon M. Büllmann, Sophia Conwell and Andres Jäschke*

Inhalt

1. General Information 1
2. Sequences 3
3. Synthesis 5
4. DNAzyme Assays 9
5. dU-Ph(^{t}Bu)$_2$ 10
6. Oligonucleotide Synthesis 11
7. UV/Vis Spectra 12
8. HPLC Analysis for PSS^{UV} Determination 13
9. Thermostability Measurements 14
10. Reversibility Measurements 15
11. PAGE Data 16
12. HPLC Analysis of the Closed Form 20
13. NMR Spectra 21
14. Supplementary References 23

1. General Information

Synthesis

Commercially available reagents were procured from Sigma Aldrich, Carbolution, and Carbosynth and employed without additional purification. Anhydrous solvents were acquired in sealed containers over molecular sieves. Solvents necessary for reactions sensitive to oxygen were degassed before use through a continuous argon flow across the reaction mixture for approximately 10 minutes. The progress of all reactions was tracked using TLC (thin-layer chromatography) on silica plates coated with a fluorescent indicator and visualized under UV light (254 nm) or stained with a blue-shift solution ($Ce(SO_4)_2$; molybdatophosphoric acid; sulfuric acid). Reactions conducted under elevated pressure occurred either in an autoclave (Büchi) equipped with a manometer or in microwave tubes sealed with a gas-tight crimp cap. Flash chromatography followed the procedure outlined by Still *et al.*[39] employing silica gel 60 (0.04-0.063 mm) and laboratory-grade solvents.

The synthesis of the phosphoramidite from dU-Me-Ph^{t}Bu was carried out after the protocol of Kolmar and Büllmann *et al.*[15] The dU-Ph^{t}Bu was synthesized as described by Büllmann *et al.*[16]

Synthesis of oligonucleotides

The unmodified 8-17 DNAzyme and the substrate strand oligonucleotide were purchased from IDT and used without further purification.

The DAE-modified oligonucleotides were synthesized via solid-phase phosphoramidite chemistry using an automated oligonucleotide synthesizer (H6, V.01.02, K&A Laborgeräte GbR) in the 5′-DMT-off mode. A standard 1 µmol DNA protocol was used, whereas dwell time (+220%) and phosphoramidite volume (+75%) was increased for the DAE-modified monomers. Reagents were sourced from Roth and Sigma-Aldrich (Proligo) and utilized without additional purification. A 500 Å Controlled Pore Glass (CPG) served as the solid support. Standard DNA phosphoramidites were dissolved in dry acetonitrile to achieve a concentration of 0.05 M, while modified phosphoramidites were dissolved to a concentration of 0.07 M.

Post-synthesis, the solid phase was treated with 25% aqueous ammonia (4 h, 40 °C) and rinsed thrice with Millipore water (1 mL) to fully extract the oligonucleotide from the solid support. The ammonia/water mixture was lyophilized, and the crude product was dissolved in water, filtered, and purified via HPLC using C18-SE column (VDSpher Pur 100 C18-SE, 5 µm, 250 × 4.6 mm, VDS Optilab with a flow rate of 1 mL/min. Solvent gradients of buffer A and buffer B (buffer A = 10 mM triethylammonium acetate (pH 7) in water, buffer B = 10 mM triethylammonium acetate (pH 7) in acetonitrile) were employed (0 min – 20 min, 10% – 22% B; 20 min – 21 min, 22% – 35% B; 21 min – 40 min, 35% – 55% B). The manually collected product fractions were lyophilized twice, and the remaining oligonucleotide was dissolved in 0.5 mL Millipore water. Concentration was determined using a Nanodrop One (Thermo Fisher Scientific), and the aqueous stock solutions were stored at –21 °C.

Analytics

High-resolution mass spectra were acquired utilizing a Bruker microTOF-Q II ESI (ESI). NMR spectra were obtained using Varian Systems 300 and 500 instruments. Chemical shifts (δ) are denoted in parts per million (ppm), referenced downfield of TMS and to the corresponding residual undeuterated solvent peak: $CDCl_3$= 7.26 ppm for ^{1}H-NMR and $CDCl_3$= 77.0 ppm; for ^{13}C-NMR. Apparent coupling constants (J) are represented in Hz. HPLC analyses were conducted utilizing an Agilent 1100 Series HPLC system equipped with a diode array detector (DAD). A VDS optilab C18 column (VDSpher OptiBio Pur 300 C18-H, 250 x 4.66 mm) was employed, running at a flow rate of 1 mL/min with elution via a gradient of water and acetonitrile or buffer A and B (buffer A = 10 mM triethylammonium acetate (pH 7) in water, buffer B = 10 mM triethylammonium acetate (pH 7) in acetonitrile).

Photochromic Properties

For the measurement of oligonucleotide absorbance spectra, a 400 µL solution containing 30 µM of the sample in water was prepared and transferred into a Starna quartz cuvette (Model: 28-F-Q). The sample was subsequently exposed to UV-light LED irradiation (340 nm LED: Thorlabs, Mounted High Power LED, operated at 700 mA) at specific time intervals. An absorbance spectrum was then acquired using a Cary 100 Bio UV/VIS spectrometer by Varian, and the resulting data were analyzed utilizing CaryWinUV and OriginPro 15 software.

Photostationary state (PSS)

For the evaluation of the switching efficiency, HPLC analyses were conducted utilizing an Agilent 1100 Series HPLC system that was equipped with a diode array detector (DAD). The VDS optilab C18 column (VDSpher OptiBio Pur 300 C18-H, 250 x 4.66 mm) was employed, running at a flow rate of 1.0 mL/min and employing a gradient elution with buffer A (10 mM triethylammonium acetate in water, pH 7) and buffer B (10 mM triethylammonium acetate in acetonitrile, pH 7). Detection was carried out at the isosbestic point (260 nm) representing equal extinction coefficients of the open and closed isomers of the photoswitch. The switching efficiency was determined by calculating the ratio of peak areas corresponding to the open and closed isomers at wavelengths corresponding to the isosbestic point. Peaks that did not achieve complete separation via HPLC were isolated using the peak analyzer of OriginPro 2015.

Thermal stability measurements

For the assessment of the thermal stability of the oligonucleotides, a 400 µL solution comprising 30 µM of the sample in water was added to a quartz cuvette from Starna (Model: 28-F-Q). The sample underwent irradiation using UV-light LEDs (340 nm LED: Thorlabs, Mounted High Power LED, operated at 700 mA) until achieving the photostationary state (PSS^{UV}). Subsequently, the absorption maximum of the closed-ring isomer at ~480 nm was monitored for 60 min at temperatures of 25 °C, 50 °C, and 70 °C using a Cary 100 Bio UV/VIS spectrometer by Varian. The obtained data underwent analysis via Cary Win UV and OriginPro 15 software, encompassing baseline correction and the plotting of the closed isomer's absorbance at 480 nm over time (A) divided by the initial absorption (A_o) against time.

Reversibility measurements

To evaluate the reversibility, a 30 µM aqueous solution of the sample (400 µL) was prepared and loaded into a quartz cuvette (Model: 28-F-Q, Starna). The solution was irradiated with 320 nm ultraviolet (UV) light using a 300 W xenon lamp (Max-303, Asahi Spectra) equipped with a high-transmission bandpass filter (XHQA320), until the photostationary state was achieved. An absorption spectrum was subsequently recorded using a Cary 50 Bio UV-Vis spectrophotometer (Varian). The sample was then exposed to 450 nm visible light from the same lamp, utilizing the XPBA450 bandpass filter, until the absorption band in the visible range was eliminated. Another absorption spectrum was then recorded. This irradiation cycle was repeated 40 times, and the absorbance peak corresponding to the closed-ring isomer in the visible region was monitored and plotted for each cycle.

Quantum yield measurements for cyclization and cycloreversion reactions

Quantum yields were determined using an updated Quantum Yield Determination Setup (QYDS) developed by Megerle *et al.*[40]. Photoswitches were irradiated utilizing UV-light LED (Seoul Viosys mounted 340 nm LED, model: CUD4AF1B) and visible light LED (Osram Oslon SSL80 470 nm LED) The output radiant power of the LEDs was calibrated against the solar cell output voltage using a Coherent power meter (model: PowerMax-USB PS19Q). Raw data obtained from the QYDS was processed using a Mathcad script provided by the Riedle group (LMU Munich). Quantum yield calculations were based on the "initial slope method," necessitating adjustment of LED power to ensure linear product formation. Data processing with the Mathcad script encompassed baseline correction,

spectral decomposition into substrate and product basis spectra, and quantum yield calculation by considering incoming photons per second and wavelength. The conversion to the cyclization product involved utilizing the extinction coefficients of both isomers. The spectral composition of the photostationary state (PSS^{UV}) was obtained via HPLC separation of isomers and peak integration at an isosbestic point, facilitating the computation of the spectrum of the pure closed isomer. The equation utilized for this computation is

$$E_{CF} = \frac{(E_{PSS} - E_{OF}) * X}{\text{Y}}$$

where X and Y denote the portions of the open and closed forms, respectively, within the PSS^{UV}. During the measurement, a "shutter file" was generated, documenting precise irradiation time points and absorbed radiant power at each step.

2. Sequences

Table S1. Sequences and masses of oligonucleotides used in this study. The purity was confirmed via HPLC and PAGE.

Variant	Sequence	Mass HR-ESI$^-$	
		Calc.	Found
8-17 DNAzyme	5'-TGA GAA GTG TCA GCG ACT CGA AAA GGA TCG-3'	9328.6178-	9328.5822
2.1-Ph^{t}Bu	5'-TGA GAA G**U**$^{\mathbf{DAE}}$G TCA GCG ACA CGA AAA GGA TCG-3'	9661.7476	9661.5959
2.1-Ph(^{t}Bu)$_2$	5'-TGA GAA G**U**$^{\mathbf{DAE}}$G TCA GCG ACA CGA AAA GGA TCG-3'	9758.7978	9758.6241
2.1-Me-Ph^{t}Bu	5'-TGA GAA G**U**$^{\mathbf{DAE}}$G TCA GCG ACA CGA AAA GGA TCG-3'	9676.6124	9675.7633
4-Ph^{t}Bu	5'-TGA GAA GTG **U**$^{\mathbf{DAE}}$CA GCG ACA CGA AAA GGA TCG-3'	9652.7361	9653.1110
8-Ph^{t}Bu	5'-TGA GAA GTG TCA G**U**$^{\mathbf{DAE}}$G ACA CGA AAA GGA TCG-3'	9676.7473	9676.6572
12-Ph^{t}Bu	5'-TGA GAA GTG TCA GCG AC**U**$^{\mathbf{DAE}}$ CGA AAA GGA TCG-3'	9652.7361	9652.1776
15-Ph^{t}Bu	5'-TGA GAA GTG TCA GCG ACT CG**U**$^{\mathbf{DAE}}$ AAA GGA TCG-3'	9643.7246	9643.7342
16-Ph^{t}Bu	5'-TGA GAA GTG TCA GCG ACT CGA **U**$^{\mathbf{DAE}}$AA GGA TCG-3'	9643.7246	9643.7058
Substrate	5'-CGA TCC TT**rG** GCT TCT CA-3'	5127.8718	5127.8373
eGFP-DNAzyme1	5’-GTA GCG G**U**$^{\mathbf{DAE}}$G TCA GCG ACA CGA AGA AGC ACT-3’	9607.8124	9607.9701
eGFP-DNAzyme2	5’-GTG CGC T**U**$^{\mathbf{DAE}}$G TCA GCG ACA CGA ATG GAC GTA-3’	9595.7022	9595.7764
Substrate eGFP-DNAzyme1	5’-AGU GCU UCrA GCC GCU AC-3’	5161.8898	5161.8633
Substrate eGFP-DNAzyme2	5’-UAC GUC CArG GAG CGC AC-3’	5195.9077	5195.8736

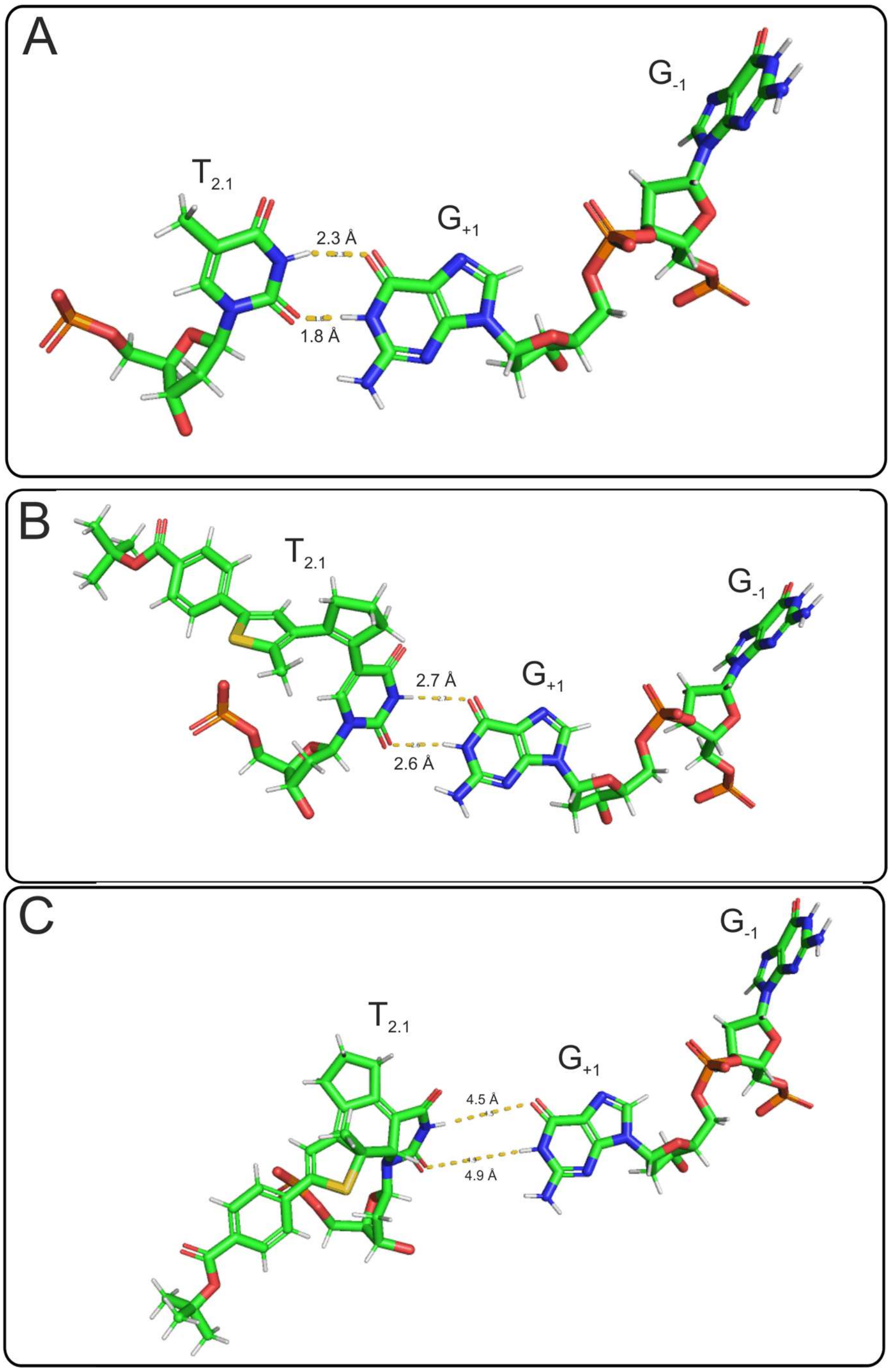

Figure S1. Proposed impact of dU-Ph^{t}Bu DAE OF and CF on the T2.1:G+1 base pair. A) Geometry and H-bonding distances as observed in the original crystal structure (PDB: 8or8).[21] B, C) T2.1 was modified in PyMOL to resemble the OF (B) or CF (C) of 2.1-Ph^{t}Bu and subsequently subjected to geometric optimization (Kabsch algorithm).

3. Synthesis

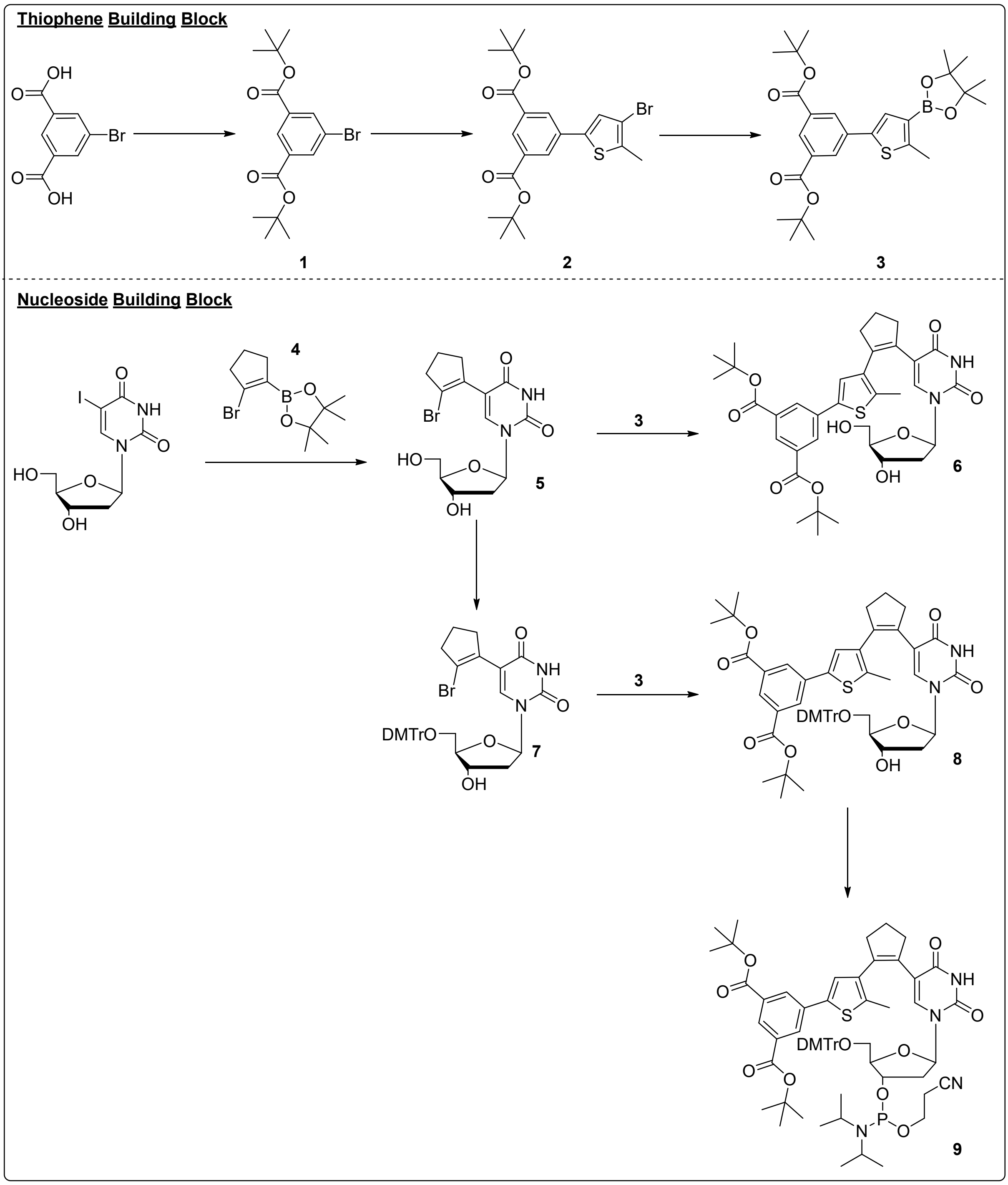

Scheme S1: Synthesis scheme for the newly developed dU-(Ph^{t}Bu)$_2$ as nucleoside (**6**) and as DMT protected phosphoramidite (**9**).

Di-tert-butyl 5-bromoisophthalate (1)

5-Bromoisophthalic acid (1.00 g, 4.08 mmol, 1 equiv.) and potassium tert-butoxide were placed in an oven-dried Schlenk flask. The atmosphere was purged and replaced with argon three times. Thionyl chloride (9.71 g, 81.62 mmol, 20 equiv.) and catalytic amount of DMF were then added. The reaction mixture was stirred at 75 °C for 1 hour. Subsequently, thionyl chloride was removed under vacuum and collected in a freeze trap. The resulting solid residue was dissolved in DCM and cooled to 0 °C. Potassium tert-butoxide (503.76 mg, 4.49 mmol, 1.1 equiv.) was then added slowly, and the mixture

was stirred at room temperature for 1 hour. The reaction mixture was quenched with ice water, and the aqueous phase was extracted with DCM. The combined organic extracts were dried over $MgSO_4$ and concentrated. The crude product was purified via silica column chromatography using a DCM–EtOAc gradient (0–8% EtOAc). The purification yielded compound (1) as a colorless solid (680 mg, 1.90 mmol, 47%). The spectral data matches the ones in literature.[5]

^{1}H NMR (300 MHz, Chloroform-*d*) δ 8.49 (t, *J* = 1.5 Hz, 1H), 8.24 (d, *J* = 1.5 Hz, 2H), 1.60 (s, 18H).

Di-tert-butyl 5-(4-bromo-5-methylthiophen-2-yl)isophthalate (2)

Compound **1** (680 mg, 1.90 mmol, 1 equiv.), sodium carbonate (605.23 mg, 5.71 mmol, 3 equiv.), palladium-tetrakis(triphenylphosphine) (109.98 mg, 95.17 µmol, 0.05 equiv.) and (4-bromo-5-methylthiophen-2-yl)boronic acid (505 mg, 2.28 mmol, 1.20 equiv.) were presented in a flask. The atmosphere was changed three times with argon. As solvents a 5:1 mixture of THF/Water was degassed for 15 min and then added to the flask. The reaction was stirred for 18 h at 85 °C. The Mixture was quenched with brine and extracted with EtOAc. The organic phase was dried over $MgSO_4$ and concentrated *in vacuo*. The crude compound was purified with a silica column chromatography using a Cyclohexane – EtOAc gradient (0-10% EtOAc). The purification yielded **2** (400 mg, 0.882 mmol, 46%) as an off white solid.

^{1}H NMR (300 MHz, Chloroform-*d*) δ 8.45 (t, *J* = 1.5 Hz, 1H), 8.25 (d, *J* = 1.5 Hz, 2H), 7.22 (s, 1H), 2.44 (s, 3H), 1.60 (s, 18H).

^{13}C NMR (75 MHz, Chloroform-*d*) δ 164.82, 139.36, 135.02, 134.02, 133.16, 129.75, 129.40, 126.86, 110.33, 82.07, 77.16, 28.31.

Di-tert-butyl 5-(5-methyl-4-(4,4,5,5-tetramethyl-1,3,2-dioxaborolan-2-yl)thiophen-2-yl)isophthalate (3)

Compound **2** (400 mg, 882 µmol, 1 equiv.), potassium acetate (260 mg, 2.65 mmol, 3 equiv.), palladium-tetrakis(triphenylphosphine) (50.98 mg, 44.11 µmol, 0.05 equiv.), 2-Dicyclohexylphosphin-2′,4′,6′-triisopropylbiphenyl (42.1 mg, 88.22 µmol, 0.10 equiv.) and 4,4,4',4',5,5,5',5'-octamethyl-2,2'-bi(1,3,2-dioxaborolane) (291 mg, 1.15 mmol, 1.30 equiv.) were presented in an oven dried flask. The atmosphere was changed three times with argon than dry dioxane was added. The reaction was stirred at 120 °C for 16 h. The reaction was quenched with water and extracted with EtOAc. The organic phase was dried over $MgSO_4$ and concentrated *in vacuo*. The crude compound was purified with silica column chromatography using Cyclohexane – EtOAc gradient (1-3% EtOAc). The purification yielded **3** (420 mg, 839 µmol, 95.13%)

^{1}H NMR (300 MHz, Chloroform-*d*) δ 8.40 (t, *J* = 1.5 Hz, 1H), 8.31 (d, *J* = 1.6 Hz, 2H), 7.55 (s, 1H), 2.71 (s, 3H), 1.62 (s, 18H), 1.34(s, 13H).

^{13}C NMR (75 MHz, Chloroform-*d*) δ 164.27, 153.35, 138.24, 135.56, 132.76, 130.23, 129.93, 128.44, 83.39, 81.67, 29.69, 29.64, 29.35, 28.16, 24.88, 22.64, 15.95.

HR-ESI$^+$: m/z calculated for $[C_{21}H_{25}BrNaO_4S]^+$: 523,2295; found: 523,2301.

2-(2-bromocyclopent-1-en-1-yl)-4,4,5,5-tetramethyl-1,3,2-dioxaborolane (4)

In a oven dried Schlenk flask 1,2-dibromocyclopent-1-ene (4.80 g, 21.3 mmol, 1 equiv.) and 2-isopropoxy-4,4,5,5-tetramethyl-1,3,2-dioxaborolane (5.64 mL, 27.6 mmol, 1.3 equiv.) were dissolved in dry THF. The mixtrure was cooled to -78 °C. After reaching -78 °C 2.5 M *n*-BuLi (11.1 mL, 27.6 mmol, 1.3 equiv.) was added dropwise over a time period of 45 min. The reaction was stirred for 12 h at room

temperature. The reaction was quenched with water and extracted with EtOAc. The organic phase was dried over $MgSO_4$ and concentrated *in vacuo.* The crude product was purified via silica column chromatography with a gradient of cyclohexane – EtOAc (0-5% EtOAc).

^{1}H NMR (300 MHz, Chloroform-*d*) δ 2.69 (ddt, *J* = 8.0, 7.1, 2.5 Hz, 2H), 2.46 (tt, *J* = 8.3, 2.4 Hz, 2H), 2.00 – 1.84 (m, 2H), 1.29 (s, *J* = 1.0 Hz, 12H).

5-(2-bromocyclopent-1-en-1-yl)-1-((2R,4S,5R)-4-hydroxy-5-(hydroxymethyl)tetrahydrofuran-2-yl)pyrimidine-2,4(1H,3H)-dione (5)

Compound **5** was synthesized according to C.M. Hendrich *et al*.[41]

Di-tert-butyl 5-(4-(2-(1-((2R,4S,5R)-4-hydroxy-5-(hydroxymethyl)tetrahydrofuran-2-yl)-2,4-dioxo-1,2,3,4-tetrahydropyrimidin-5-yl)cyclopent-1-en-1-yl)-5-methylthiophen-2-yl)isophthalate (6)

Compound **5** (35 mg, 93.8 µmol, 1 equiv.), sodium carbonate (29.8 mg, 281 µmol, 3 equiv.), palladium-tetrakis(triphenylphosphine) (5.4 mg, 14.8 µmol, 0.05 equi) and compound **3** (56.3 mg, 113 µmol, 1,2 equiv.) were presented in an oven dried flask and the atmosphere was changed three times with argon. The degassed solvent mixture of dimethoxyethan – water 4/1 was added and stirred for 16 h at 85 °C. Subsequently the reaction was quenched with water and extracted with EtOAc. The organic phase was dried over $MgSO_4$ and concentrated *in vacuo.* The crude product was purified via silica column chromatography with a gradient of chloroform – methanol + 0,2% triethylamine (1-3% methanol). The purification yielded **6** (180 mg, 186 µmol, 63%) as colorless solid.

^{1}H NMR (300 MHz, DMSO-*d*$_6$) δ 11.37 (s, 1H), 8.24 (d, *J* = 1.5 Hz, 1H), 8.21 (d, *J* = 1.6 Hz, 2H), 7.39 (d, *J* = 1.6 Hz, 2H), 6.06 (dd, *J* = 7.9, 5.8 Hz, 1H), 5.05 (d, *J* = 4.2 Hz, 1H), 4.79 (t, *J* = 5.0 Hz, 1H), 3.85 (d, *J* = 5.8 Hz, 1H), 3.61 (q, *J* = 3.5 Hz, 1H), 3.24 (t, *J* = 4.6 Hz, 2H), 3.06 (q, *J* = 7.2 Hz, 3H), 2.90 (q, *J* = 8.1, 7.7 Hz, 1H), 2.85 – 2.69 (m, 1H), 2.65 (s, 2H), 2.14 (s, 3H), 1.97 (dd, *J* = 8.9, 6.1 Hz, 2H), 1.58 (s, 18H).

^{13}C NMR (75 MHz, DMSO-*d*$_6$) δ 163.88, 163.81, 161.98, 149.79, 137.83, 136.97, 136.46, 134.87, 134.73, 134.43, 133.36, 132.53, 129.01, 127.65, 126.50, 111.12, 87.35, 83.61, 81.80, 81.70, 70.67, 61.40, 45.56, 37.45, 35.77, 27.70, 13.87, 8.66.

HR-ESI$^+$: m/z calculated for $[C_{35}H_{42}NaN_2O_9S]^+$ 689.2503; found 689.2495

1-((2R,4S,5R)-5-((bis(4-methoxyphenyl)(phenyl)methoxy)methyl)-4-hydroxytetrahydrofuran-2-yl)-5-(2-bromocyclopent-1-en-1-yl)pyrimidine-2,4(1H,3H)-dione (7)

Compound **5** (900 mg, 2.41 mmol, 1 equiv.) and 4-dimethyalminopyridine (14.7 mg, 121 µmol, 0,05 equiv.) were presented in an oven dried flask. The atmosphere was changed three times. Dry pyridine was added at 0 °C. DMT-Cl (937 mg, 2.77 mmol, 1.15 equiv.) was presented in oven dried flask and solved in dry pyridine. The solution of DMT-Cl was then added to the solution of compound **5** over a time period of 30 min. The reaction was stirred overnight at room temperature. The reaction was quenched with MeOH and concentrated *in vacuo*. The crude compound was purified with silica column chromatography using DCM – MeOH (1-5% MeOH). The purification yielded **7** (1.3 g, 1.29 mmol, 80%)

^{1}H NMR (500 MHz, Methanol-d4) δ 7.75 (s, 1H), 7.42 (d, J = 8.7 Hz, 2H), 7.32 – 7.28 (m, 5H), 7.23 – 7.17 (m, 2H), 6.85 (d, J = 9.0 Hz, 4H), 6.31 – 6.26 (m, 1H), 4.39 (dt, J = 6.7, 3.6 Hz, 1H), 4.05 (t, J = 3.7 Hz, 1H), 3.78 (s, 6H), 2.57 (d, J = 5.6 Hz, 2H), 2.44 – 2.36 (m, 3H), 2.29 – 2.25 (m, 1H), 1.83 – 1.72 (m, 2H).

^{13}C NMR (126 MHz, Methanol-d4) δ 163.87, 160.20, 151.65, 150.07, 146.14, 140.19, 137.12, 137.01, 135.10, 131.29, 129.34, 128.87, 127.51, 121.58, 114.19, 111.66, 87.91, 87.01, 72.51, 65.13, 55.72, 41.73, 41.42, 35.83, 22.92.

HR-ESI$^+$: m/z calculated for $[C_{35}H_{35}BrNaN_2O_7]^+$: 697.1520; found 697.1514

Di-tert-butyl 5-(4-(2-(1-((2R,4S,5R)-5-((bis(4-methoxyphenyl)(phenyl)methoxy)methyl)-4-hydroxytetrahydrofuran-2-yl)-2,4-dioxo-1,2,3,4-tetrahydropyrimidin-5-yl)cyclopent-1-en-1-yl)-5-methylthiophen-2-yl)isophthalate (8)

Compound **7** (200 mg, 296 µmol, 1 equiv.), sodium carbonate (94,2 mg, 888 µmol, 3 equiv.), palladium-tetrakis(triphenylphosphine) (17.1 mg, 14.8 µmol, 0.05 equiv.) and compound **3** (200 mg, 400 µmol, 1,35 equiv.) were presented in an oven dried flask and the atmosphere was changed three times with argon. The degassed solvent mixture of dimethoxyethan – water 4/1 was added and stirred for 16 h at 85 °C. Subsequently the reaction was quenched with water and extracted with EtOAc. The organic phase was dried over MgSO4 and concentrated *in vacuo.* The crude product was purified via silica column chromatography with a gradient of chloroform – methanol + 0,2% triethylamine (1-3% methanol). The purification yielded **8** (180 mg, 186 µmol, 63%) as colorless solid.

^{1}H NMR (500 MHz, Chloroform-*d*) δ 8.40 (t, *J* = 1.6 Hz, 1H), 8.14 (d, *J* = 1.6 Hz, 2H), 7.36 – 7.30 (m, 2H), 7.29 – 7.14 (m, 8H), 6.91 (s, 1H), 6.84 – 6.73 (m, 5H), 6.17 (dd, *J* = 7.0, 5.9 Hz, 1H), 3.92 (dt, *J* = 7.6, 5.3 Hz, 1H), 3.79 (d, *J* = 4.0 Hz, 6H), 3.22 – 3.05 (m, 2H), 2.82 – 2.68 (m, 1H), 2.68 – 2.57 (m, 1H), 2.56 – 2.39 (m, 2H), 2.16 (ddd, *J* = 13.9, 7.1, 5.3 Hz, 1H), 2.08 (s, 3H), 1.61 (s, 18H), .

^{13}C NMR (126 MHz, Chloroform-*d*) δ 165.06, 161.65, 158.73, 158.67, 149.51, 144.61, 138.92, 137.58, 136.82, 136.54, 135.95, 135.59, 135.19, 134.89, 133.51, 133.09, 130.25, 130.21, 130.00, 129.03, 128.26, 128.05, 127.00, 125.80, 113.32, 112.38, 86.55, 84.54, 83.68, 82.30, 71.59, 64.00, 55.35, 40.44, 38.02, 36.08, 28.29, 14.18.

HR-ESI$^+$: m/z calculated for $[C_{56}H_{60}NaN_2O_{11}S]^+$:991.3810; found 991.3808

Di-tert-butyl 5-(4-(2-(1-((2R,4S,5R)-5-((bis(4-methoxyphenyl)(phenyl)methoxy)methyl)-4-(((2-cyanoethoxy)(diisopropylamino)phosphaneyl)oxy)tetrahydrofuran-2-yl)-2,4-dioxo-1,2,3,4-tetrahydropyrimidin-5-yl)cyclopent-1-en-1-yl)-5-methylthiophen-2-yl)isophthalate (9)

Compound **8** (100 mg, 103.18 µmol, 1 equiv.) was placed in a dried Schlenk flask and dissolved in 1.5 mL dry DCM and cooled to 0 °C. A mixture of 2-cyanoethyl N,N-diisopropylchlorophosphoramidite (27 µL, 119 µmol, 1.15 equiv.) and DIPEA (36 µL, 206 µmol, 2 equiv.) in 0.5 mL dry DCM was added. After 2 h stirring at 0 °C the TLC indicated complete conversion. The reaction was washed with 5% $NaHCO_3$ and extracted with DCM. The organic phase was dried over $MgSO_4$ and concentrated *in vacuo*. The product was used without any further purification for solid phase synthesis.

4. DNAzyme Assays

The 2x DNAzyme cleaving buffer contains HEPES buffer (pH 8, 100 mM), NaCl (400 mM) and $MgCl_2$ (20 mM).

The substrate (10 µM) was labelled in 1x T4 polynucleotide kinase buffer A with γ-^{32}P-ATP (3 µCi/µL) at the 5'-OH using T4 polynucleotide kinase (1 U/µL). The described mixture was incubated for 1 h at 37 °C. Subsequently, the reaction was purified with the QIAquick® Nucleotide Removal Kit. The purity was determined through 20%-PAGE analysis. The concentration was determined using a Nanodrop spectrometer.

The labelled substrate was then used in the cleavage assay to evaluate each modification incorporated into the DNAzyme. In a 1.5 mL Eppendorf tube 1x DNAzyme buffer and labelled substrate strand (20 nM) were assembled and heated to 37 °C. A 5 µL sample was withdrawn (T_0). After addition of DNAzyme (90 nM) samples were withdrawn at time points: 0.5, 1, 2, 3, 5, 7, 10, 20, and 60 minutes. Each sample taken was mixed with 5 µL of 2x Loading Dye. Subsequently, all samples were heated at 95 °C for 5 minutes and then analyzed via a 20%-PAGE, followed by phosphorimaging. This assay was conducted in triplicate for each form (OF & PSS^{UV}) of each modification. The phosphor screens were scanned using a Typhoon FLA 9500 scanner and the resulting images were processed with ImageQuant TL software (Version 8.1). The obtained values were subsequently imported into OriginPro 2015 for further analysis. The percentage of the cleaved substrate was calculated and plotted. Through a linear fit applied to the burst phase, the initial velocity (v_0) was determined.

Reversible switching of DNAzyme activity

The substrate was labelled and purified as described in the previous chapter. Afterwards the sample for the reversibility activity measurement was prepared with DNAzyme (0.1 µM) and labelled substrate (0.1 µM) excluding the DNAzyme Buffer.

The Measurement includes the following steps:

1. Heating the sample at 70 °C for 3 min.
2. Adding the DNAzyme Buffer (1x).
3. Collecting T_0 aliquot 5 µL.
4. Incubating for 7 min.
5. Heating the sample at 70 °C for 3 min.
6. Collecting T_1.
7. Irradiating with 340 nm/490 nm for 0.5 min.
8. Repeat step 4-7 four times.

The times points were T_X:0,10 ,20 ,30 ,40 and 50 min. The aliquot was directly diluted with denaturing loading dye. Subsequently, all samples were heated at 95 °C for 5 minutes and then analyzed via a 20%-PAGE, followed by phosphorimaging and analysis as described above. This assay was conducted in triplicate. Control experiments, in which the DAE remained either in OF or PSS^{UV} throughout the entire experiment, were conducted following the same procedure, omitting the irradiation steps. The two following LEDS were used: 340 nm LED, Thorlabs, Mounted High Power LED, operated at 700 mA and 490 nm LED, Thorlabs, Mounted High Power LED, operated at 350 mA.

Kinetic measurement after thermostability or reversibility

A sample of the 2.1-Ph^{t}Bu-modified strand was subjected to a single thermal treatment at 70 °C, following the procedure outlined in the “Thermal Stability Measurement” section. Another sample underwent a single treatment according to the protocol described in the “Reversibility Measurement” section. Subsequently, kinetic measurements for the open form (OF) were performed as detailed in the “Kinetic Measurements” section.

5. dU-Ph(^{t}Bu)$_2$

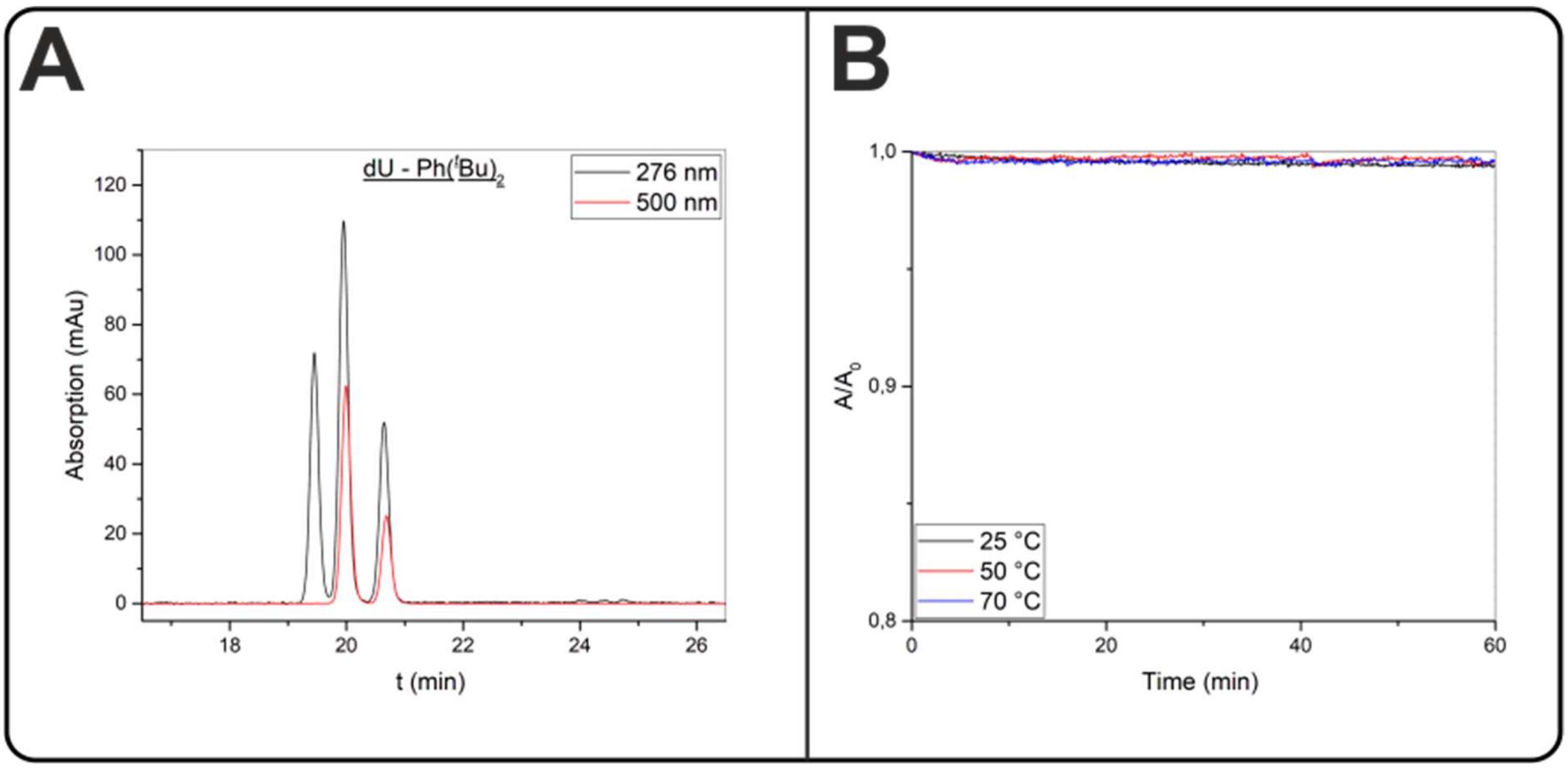

Figure S2. Additional data of photophysical properties for dU-Ph(^{t}Bu)$_2$. A) **.** Determination of the PSS^{UV}. A 60 μM solution was irradiated with UV light to the PSS^{UV} and analyzed via HPLC. The peaks were integrated at the isosbestic wavelength to calculate the PSS^{UV}. B) Thermal stability normalized to the starting value to calculate the remaining CF in %. A 60 μM solution in 4:1 water/ethanol for was irradiated in the PSS^{UV} and the absorption at the visible light absorption maximum for each derivative. The measurement was conducted under 25/50/70 °C for 1 h.

6. Oligonucleotide Synthesis

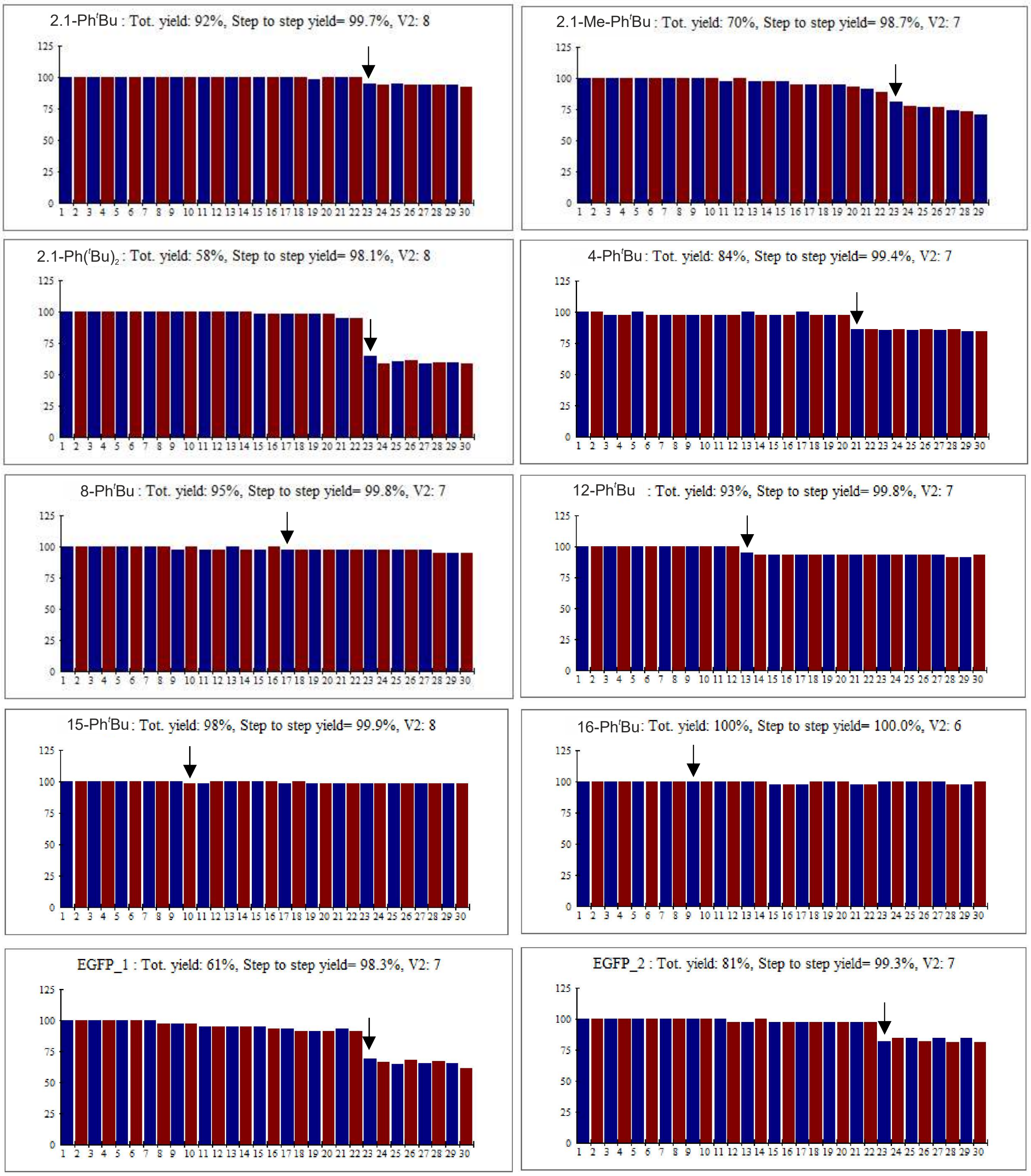

Figure S3. Trityl diagrams of the modified DNAzyme strands. Trityl absorbance is measured by the H-6 oligonucleotide synthesizer and used to calculate the step to step and the overall yield. The arrow indicates the step of incorporation of the DAE-modified monomer.

7. UV/Vis Spectra

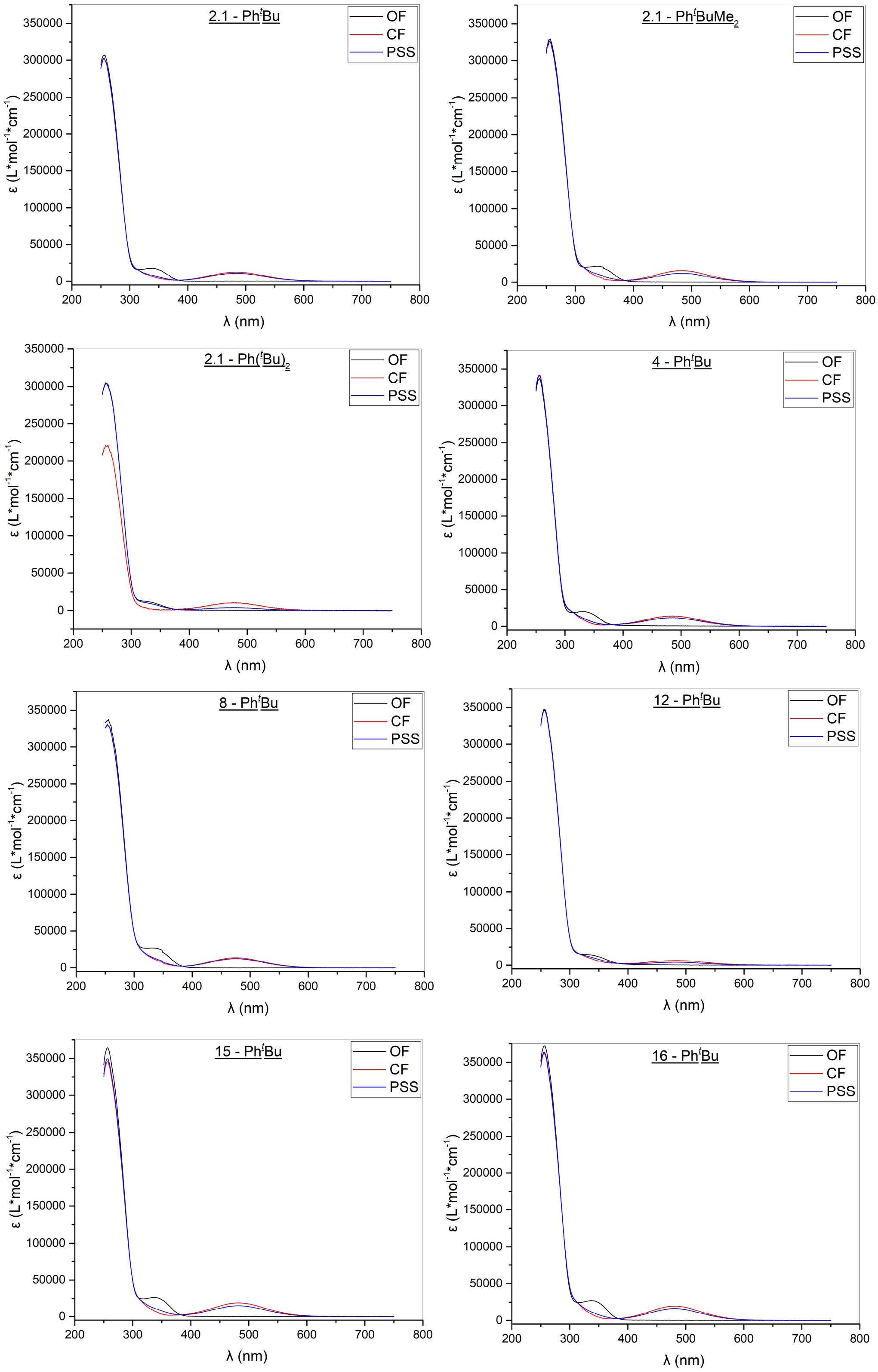

Figure S4. The UV/Vis absorption spectra of the OF, PSS^{UV} and calculated CF of the DAE compounds. The oligonucleotides were dissolved in pure water with a concentration of 30 μM.

8. HPLC Analysis for PSS^{UV} Determination

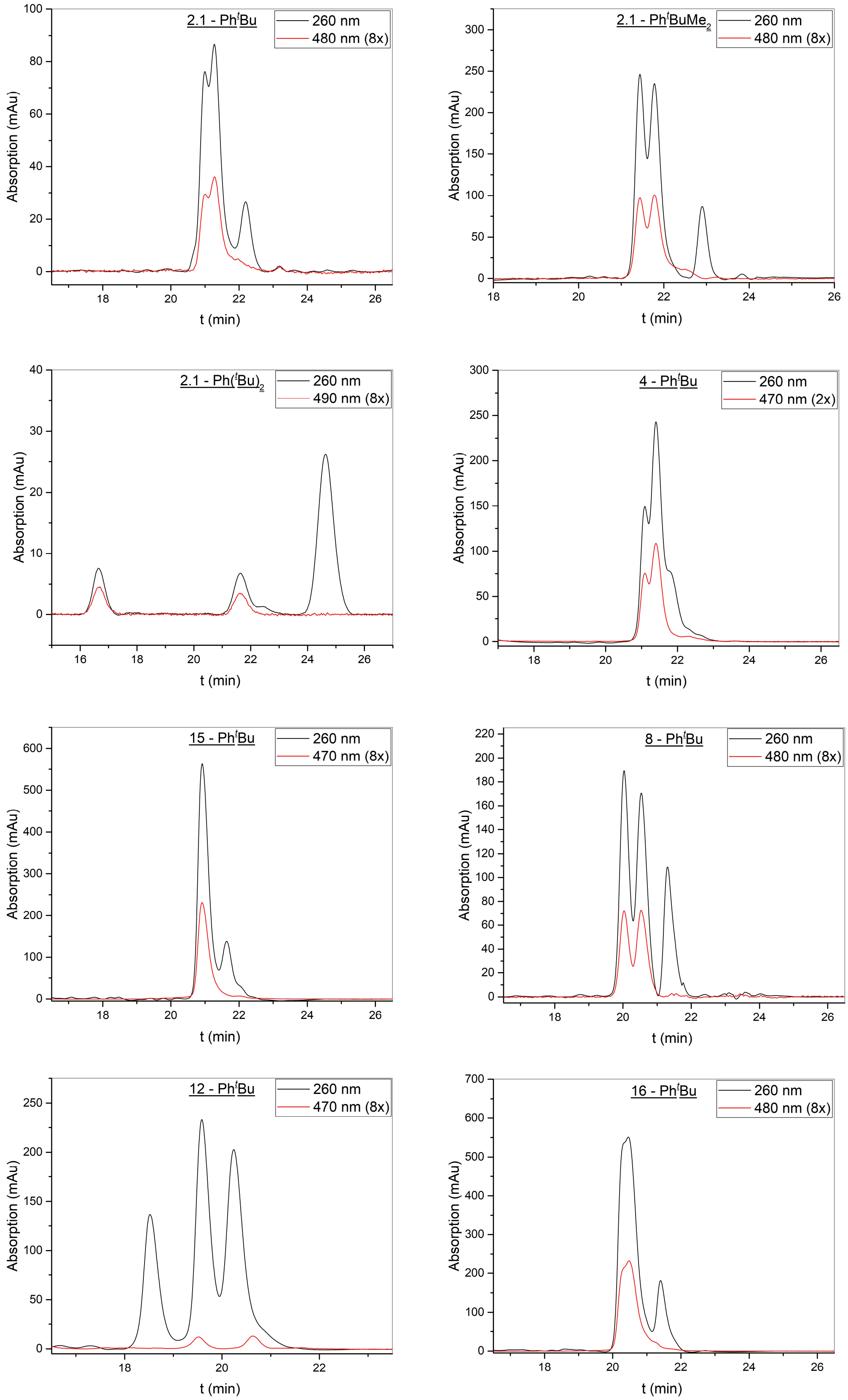

Figure S5. Determination of the PSS^{UV}. A 30 µM solution was irradiated with UV light to the PSS and analyzed via HPLC. The peaks were integrated at the isosbestic wavelength to calculate the PSS^{UV}.

9. Thermostability Measurements

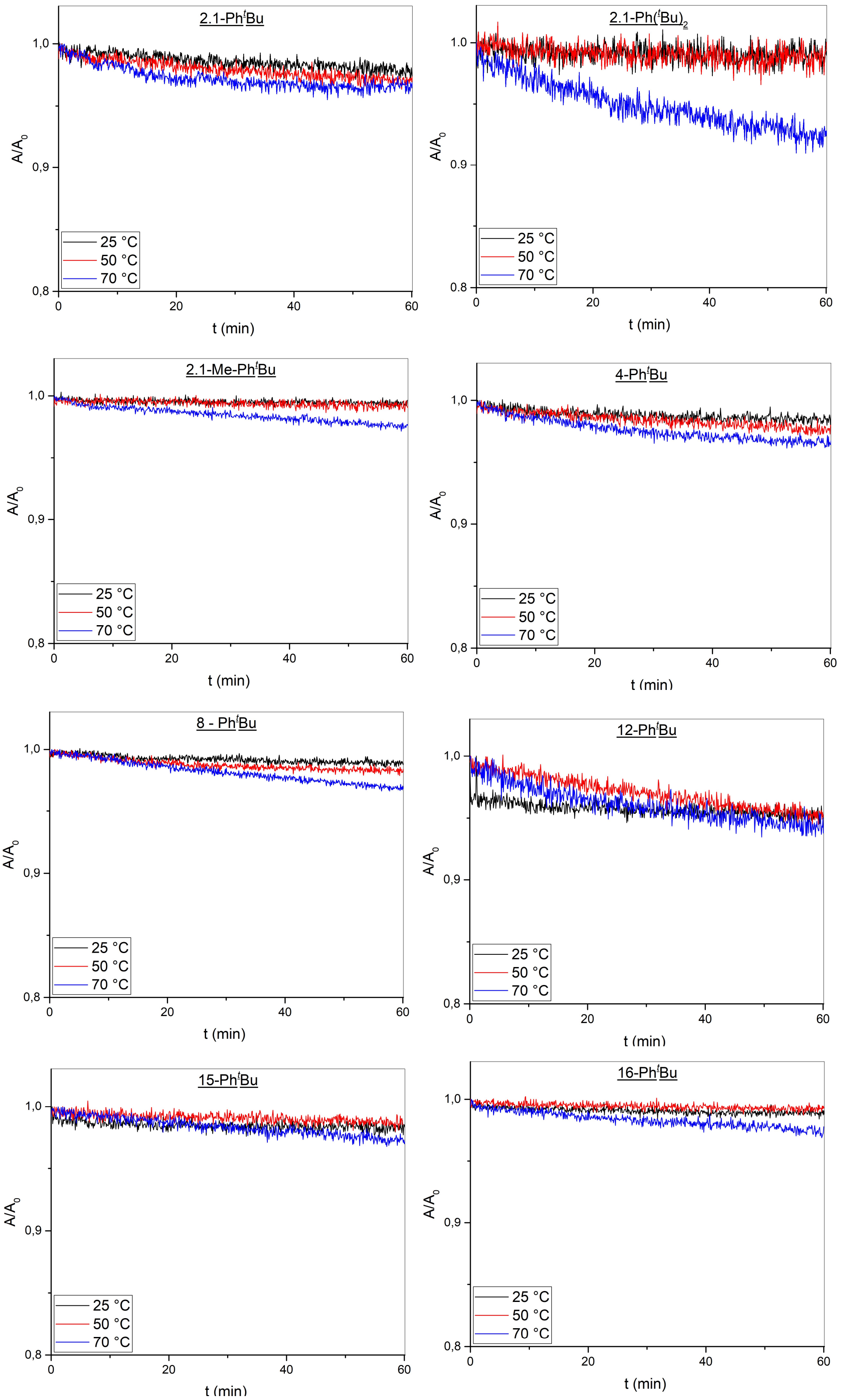

Figure S6. Thermal stability normalized to the starting value to calculate the remaining CF in %. A 30 µM solution in water (4:1 water/ethanol for dU-Ph(^{t}Bu)$_2$) was irradiated in the PSS^{UV} and the absorption at the visible light absorption maximum for each derivative. The measurement was conducted under 25/50/70 °C for 1 h.

10. Reversibility Measurements

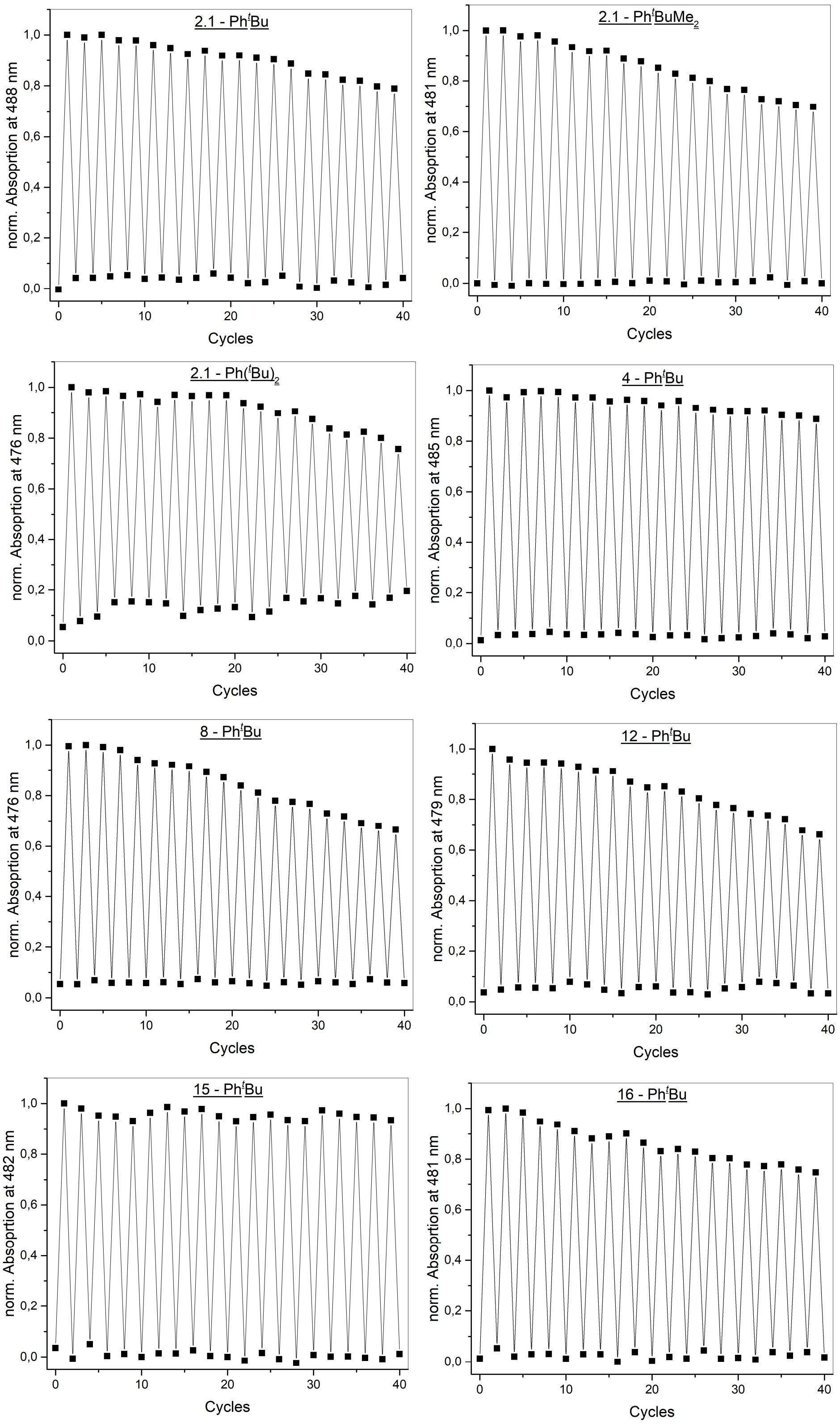

Figure S7. Reversibility measurement of every derivative. A 30 µM water solution of each oligo was first irradiated with 320 nm till the PSS^{UV} was reached. Then the UV/Vis spectrum was recorded and the sample irradiated with 450 nm and the UV/Vis spectrum was recorded. This workflow was repeated 40 times.GE Data

11. PAGE Data

Each gel image shows triplicate samples of the corresponding modification. In all cases, the upper band represents the uncleaved labelled substrate strand, whereas the lower band corresponds to the cleaved labelled substrate strand.

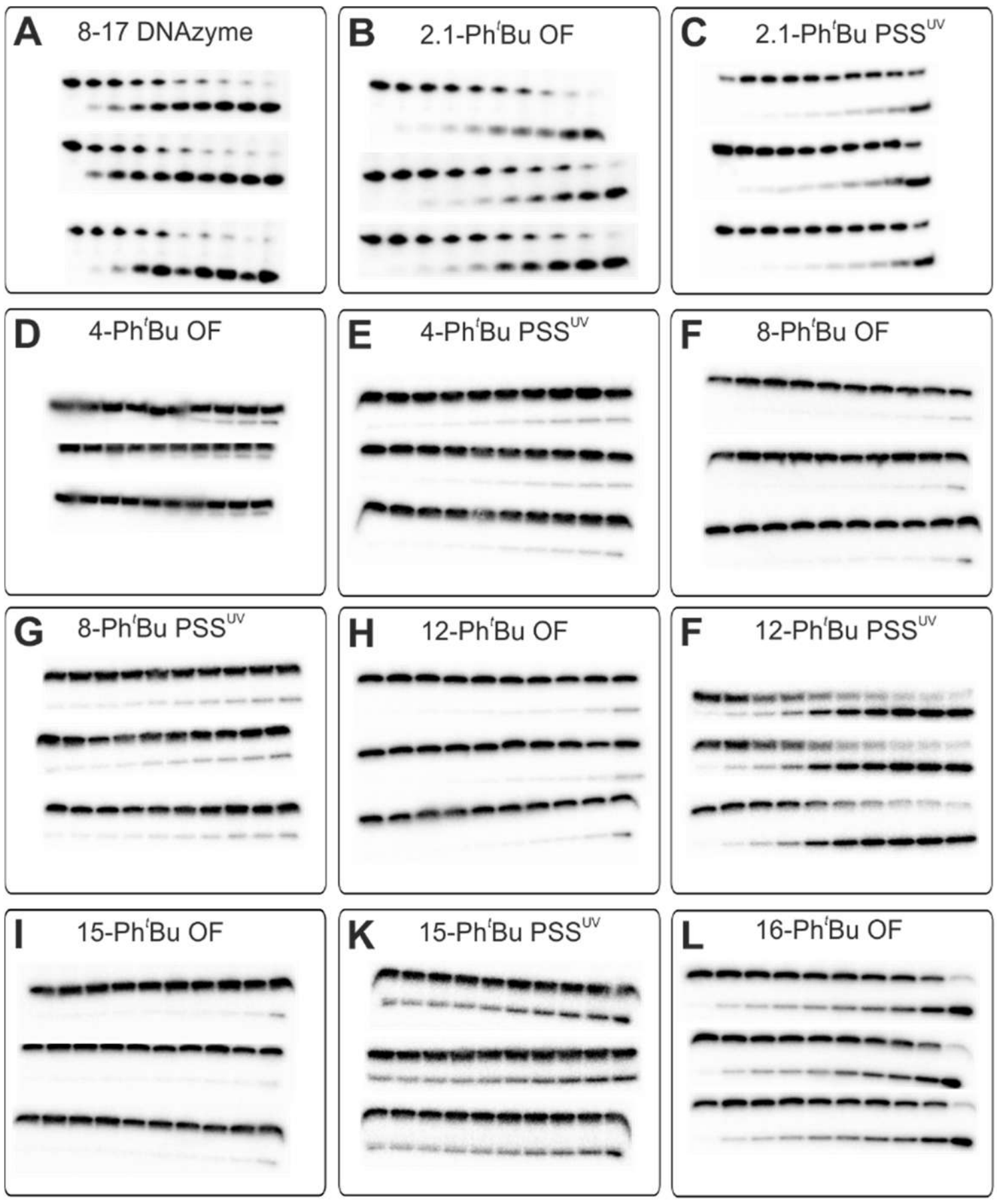

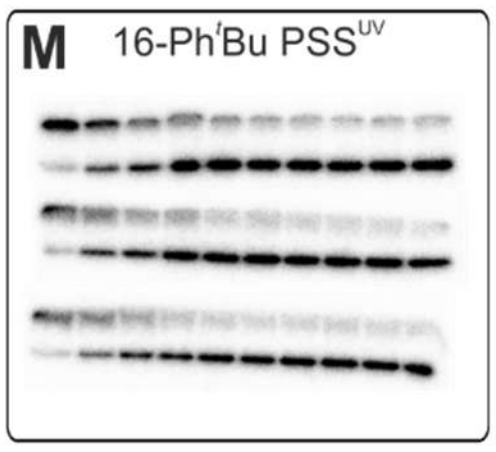

N

X_1	X_2	Velocity V_0 [%/min]	Standard deviation [%/min]
Pos	Pos	26.44633	2.34397
2.1-PhtBu	OF	12.57041	1.14519
2.1-PhtBu	PSS	1.12565	0.13179
4-PhtBu	OF	0.808	0.03349
4-PhtBu	PSS	0.498	0.00981
8-PhtBu	OF	1.653	0.00471
8-PhtBu	PSS	0.483	0.01068
12-PhtBu	OF	1.21	0.127
12-PhtBu	PSS	1.232	0.14
15-PhtBu	OF	1.28	0.331
15-PhtBu	PSS	0.502	0.01782
16-PhtBu	OF	5.419	0.271
16-PhtBu	PSS	5.291	0.874

Figure S8. Original data relating to Figure 3. A) Aliquots for the unmodified DNAzyme as positive control. Time points are: 0, 0.5, 1, 2, 3, 5, 7, 10, 20, and 60 minutes. B) Aliquots for the 2.1-Ph^{t}Bu-DNAzyme in OF. Time points are: 0, 0.5, 1, 2, 3, 5, 7, 10, 20, and 60 minutes. C) Aliquots for the 2.1-Ph^{t}Bu-DNAzyme in PSS^{UV}. Time points are: 0, 2, 6, 12, 30, 50, 70, 90, 110, 130 minutes. E) Aliquots for the 2.1-Ph^{t}Bu-DNAzyme in OF. Time points are: 0, 0.5, 1, 2, 3, 5, 7, 10, 20, and 60 minutes. D) Aliquots for the 2.1-Ph^{t}Bu-DNAzyme in PSS^{UV}. Time points are: 0, 2, 6, 12, 30, 50, 70, 90, 110, 130 minutes. F) Aliquots for the 2.1-Ph^{t}Bu-DNAzyme in OF. Time points are: 0, 0.5, 1, 2, 3, 5, 7, 10, 20, and 60 minutes. G) Aliquots for the 2.1-Ph^{t}Bu-DNAzyme in PSS^{UV}. Time points are: 0, 2, 6, 12, 30, 50, 70, 90, 110, 130 minutes. H) Aliquots for the 2.1-Ph^{t}Bu-DNAzyme in OF. Time points are: 0, 0.5, 1, 2, 3, 5, 7, 10, 20, and 60 minutes. I) Aliquots for the 2.1-Ph^{t}Bu-DNAzyme in PSS^{UV}. Time points are: 0, 2, 6, 12, 30, 50, 70, 90, 110, 130 minutes. J) Aliquots for the 2.1-Ph^{t}Bu-DNAzyme in OF. Time points are: 0, 0.5, 1, 2, 3, 5, 7, 10, 20, and 60 minutes. K) Aliquots for the 2.1-Ph^{t}Bu-DNAzyme in PSS^{UV}. Time points are: 0, 2, 6, 12, 30, 50, 70, 90, 110, 130 minutes. L) Aliquots for the 2.1-Ph^{t}Bu-DNAzyme in OF. Time points are: 0, 0.5, 1, 2, 3, 5, 7, 10, 20, and 60 minutes. M) Aliquots for the 2.1-Ph^{t}Bu-DNAzyme in PSS^{UV}. Time points are: 0, 2, 6, 12, 30, 50, 70, 90, 110, 130 minutes. N) Exact values of plotted data in Figure 3.

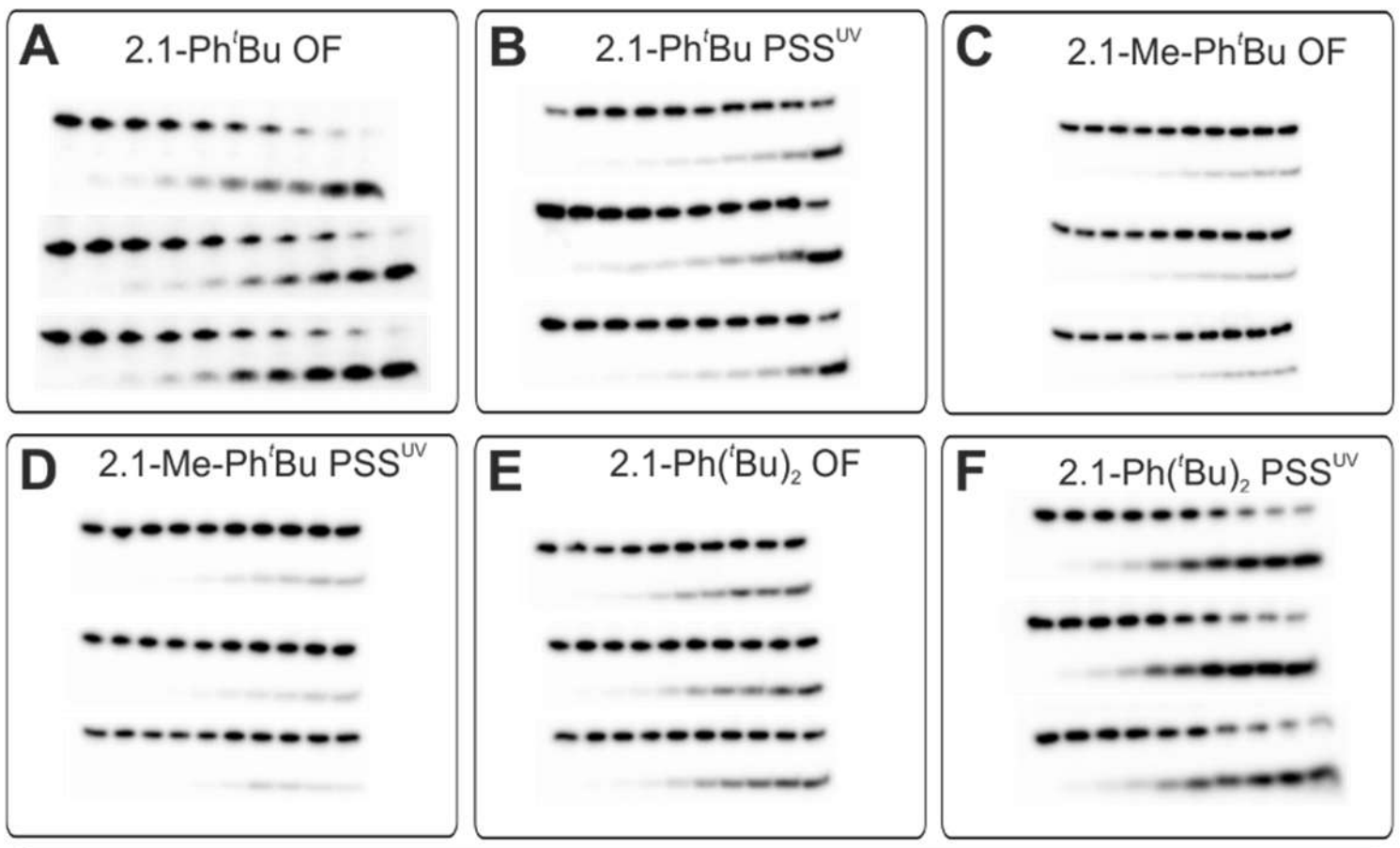

G

X_1	X_2	Velocity V_0 [%/min]	Standard deviation [%/min]
2.1 - PhtBu	OF	12.57041	1.14519
2.1 - PhtBu	PSS	1.12565	0.13179
2.1 - PhtBuMe2	OF	0.904	0.04135
2.1 - PhtBuMe2	PSS	0.953	0.0224
2.1 - Ph(tBu)2	OF	0.916	0.105
2.1 - Ph(tBu)2	PSS	1.236	0.01802

Figure S9. Original data relating to Figure 4B .A) Aliquots for the 2.1-Ph^{t}Bu-DNAzyme in OF. Time points are: 0, 0.5, 1, 2, 3, 5, 7, 10, 20, and 60 minutes. B) Aliquots for the 2.1-Ph^{t}Bu-DNAzyme in PSS^{UV}. Time points are: 0, 2, 6, 12, 30, 50, 70, 90, 110, 130 minutes. C) Aliquots for the 2.1-Me-Ph^{t}Bu-DNAzyme in OF. Time points are: 0, 2, 6, 12, 30, 50, 70, 90, 110, 130 minutes. D) Aliquots for the 2.1-Me-Ph^{t}Bu-DNAzyme in PSS^{UV}. Time points are: 0, 2, 6, 12, 30, 50, 70, 90, 110, 130 minutes. E) Aliquots for the 2.1-Ph(^{t}Bu)$_2$-DNAzyme in OF. Time points are: 0, 2, 6, 12, 30, 50, 70, 90, 110, 130 minutes. F) Aliquots for the 2.1-Ph(^{t}Bu)$_2$-DNAzyme in PSS^{UV}. Time points are: 0, 2, 6, 12, 30, 50, 70, 90, 110, 130 minutes. G) Exact values of plotted data in Figure 4B.

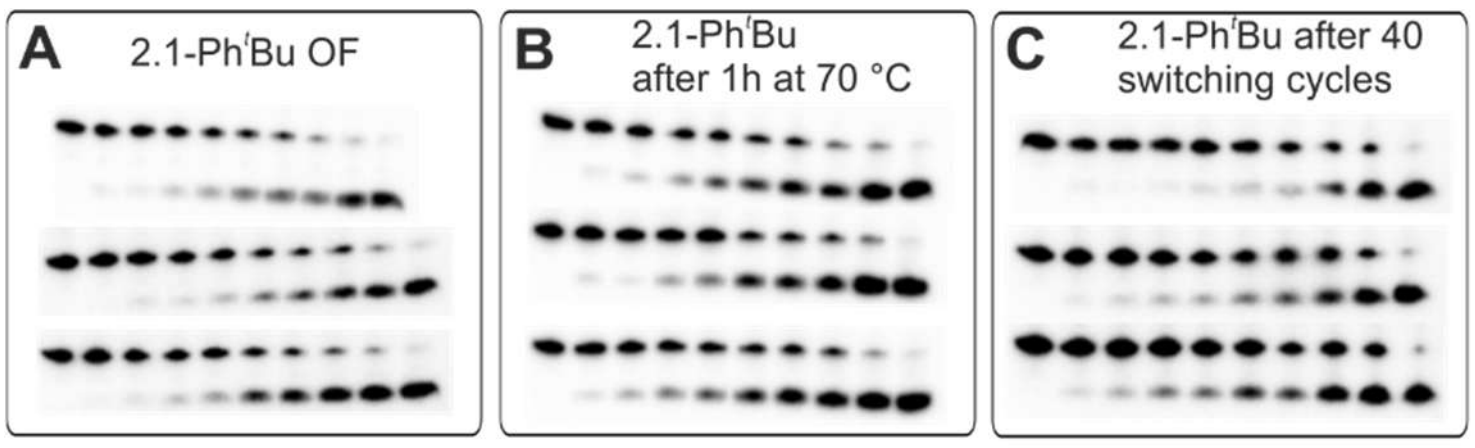

D

X_1	X_2	Velocity V_0 [%/min]	Standard deviation [%/min]
OF	Untreated	12.57041	1.14519
OF after	1 h at 70 °C	12.83733	0.05683
OF after	40 switching cycles	5.9159	0.1455
Pure	CF	0.11087	0.00723

Figure S10. Original data relating to Figure 4C. A) Aliquots for the unmodified DNAzyme as positive control. Time points are: 0, 0.5, 1, 2, 3, 5, 7, 10, 20, and 60 minutes. B) Kinetic measurement after thermostability of 2.1-Ph^{t}Bu was assessed. Time points are: 0, 0.5, 1, 2, 3, 5, 7, 10, 20, and 60 minutes. C) Kinetic measurement after reversibility 2.1-Ph^{t}Bu was assessed. Time points are: 0, 0.5, 1, 2, 3, 5, 7, 10, 20, and 60 minutes. D) Exact values of plotted data in Figure 4C.

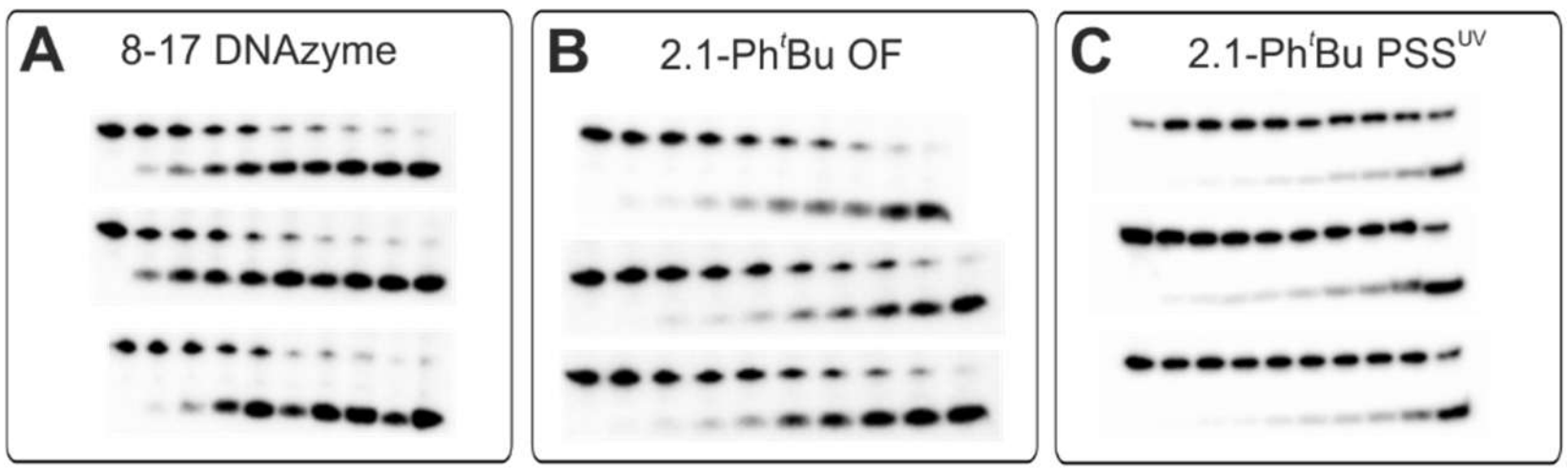

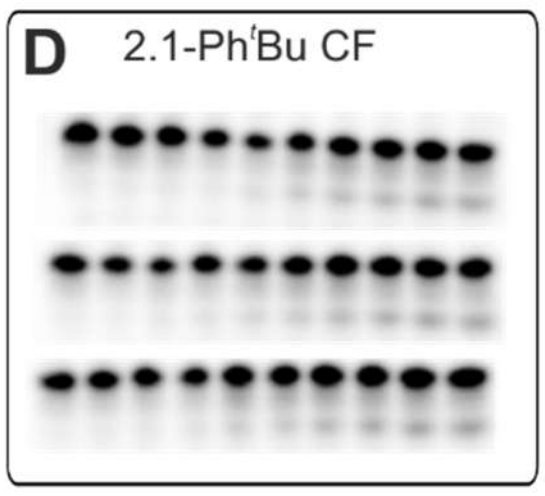

E

X_1	X_2	Velocity V_0 [%/min]	Standard deviation [%/min]
Pos	Pos	26.44633	2.34397
2.1 - PhtBu	OF	12.57041	1.14519
2.1 - PhtBu	PSS	1.12565	0.13179
2.1 - PhtBu	CF	0.06396	0.00202

Figure S11. Original data relating to Figure 4D. A) Aliquots for the unmodified DNAzyme as positive control. Time points are: 0, 0.5, 1, 2, 3, 5, 7, 10, 20, and 60 minutes. B) Aliquots for the reversible activity measurement with the 2.1-Ph^{t}Bu in the OF. Time points are: 0, 0.5, 1, 2, 3, 5, 7, 10, 20, and 60 minutes. C) Aliquots for the 2.1-Ph^{t}Bu-DNAzyme in PSS^{UV}. Time points are: 0, 2, 6, 12, 30, 50, 70, 90, 110, 130 minutes. D) Aliquots for the 2.1-Ph^{t}Bu-DNAzyme in CF. Time points are: 0, 2, 6, 12, 30, 50, 70, 90, 110, 130 minutes. E) Exact values of plotted data in Figure 4D.

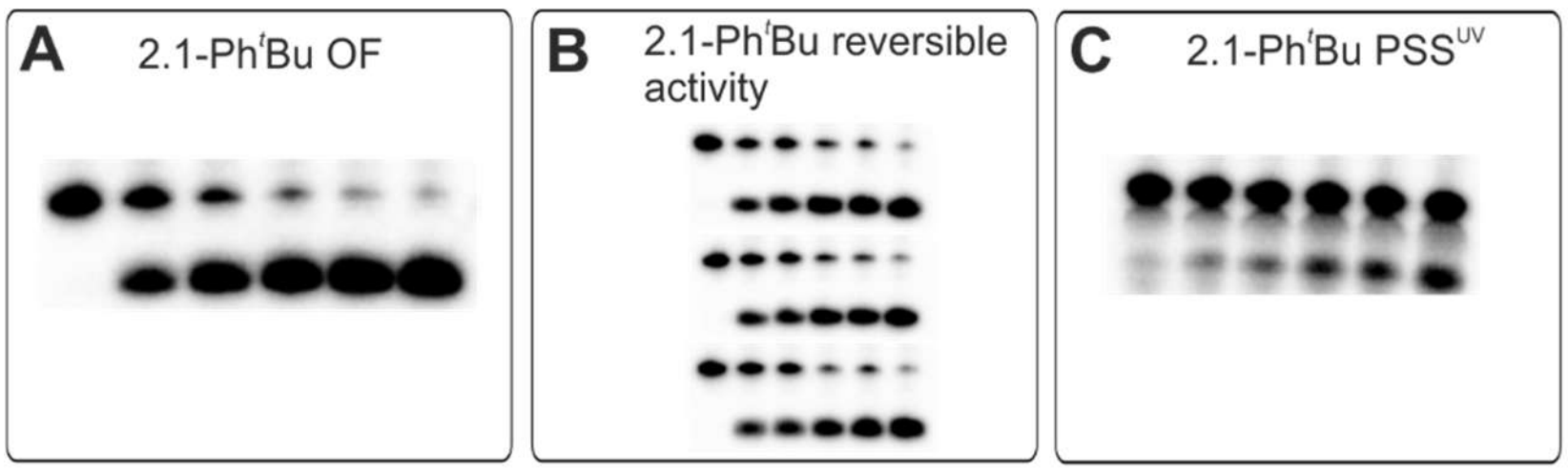

D

X	I [% cleaved]	I [% cleaved]	III [% cleaved]	Mean [% cleaved]	Standard Deviation [% cleaved]	OF [% cleaved]	PSS [% cleaved]
0	0.06459	0.04497	0.05013	0.05323	0.01017	0.01475	0.01405
10	47.22084	54.423	45.57756	49.0738	4.70484	50.19706	5.06521
20	55.55737	60.63671	54.66461	56.95289	3.22136	81.28866	7.59183
30	81.55382	84.40322	83.87513	83.27739	1.51583	92.80156	13.61196
40	84.59732	85.96893	84.10672	84.89099	0.96521	96.65154	17.9214
50	93.84843	95.03247	94.61589	94.49893	0.60062	98.02438	21.33745

Figure S12. Original data relating to Figure 4E. A) Aliquots for the reversible activity measurement with the 2.1-Ph^{t}Bu in the OF. B) Aliquots for the reversible activity measurement in which the 2.1-Ph^{t}Bu was first in OF, irradiated to PSS^{UV}, irradiated to OF, irradiated to PSS^{UV}, irradiated to OF. C) Aliquots for the reversible activity measurement with the 2.1-Ph^{t}Bu in the PSS^{UV}. D) Exact values of plotted data in Figure 4E.

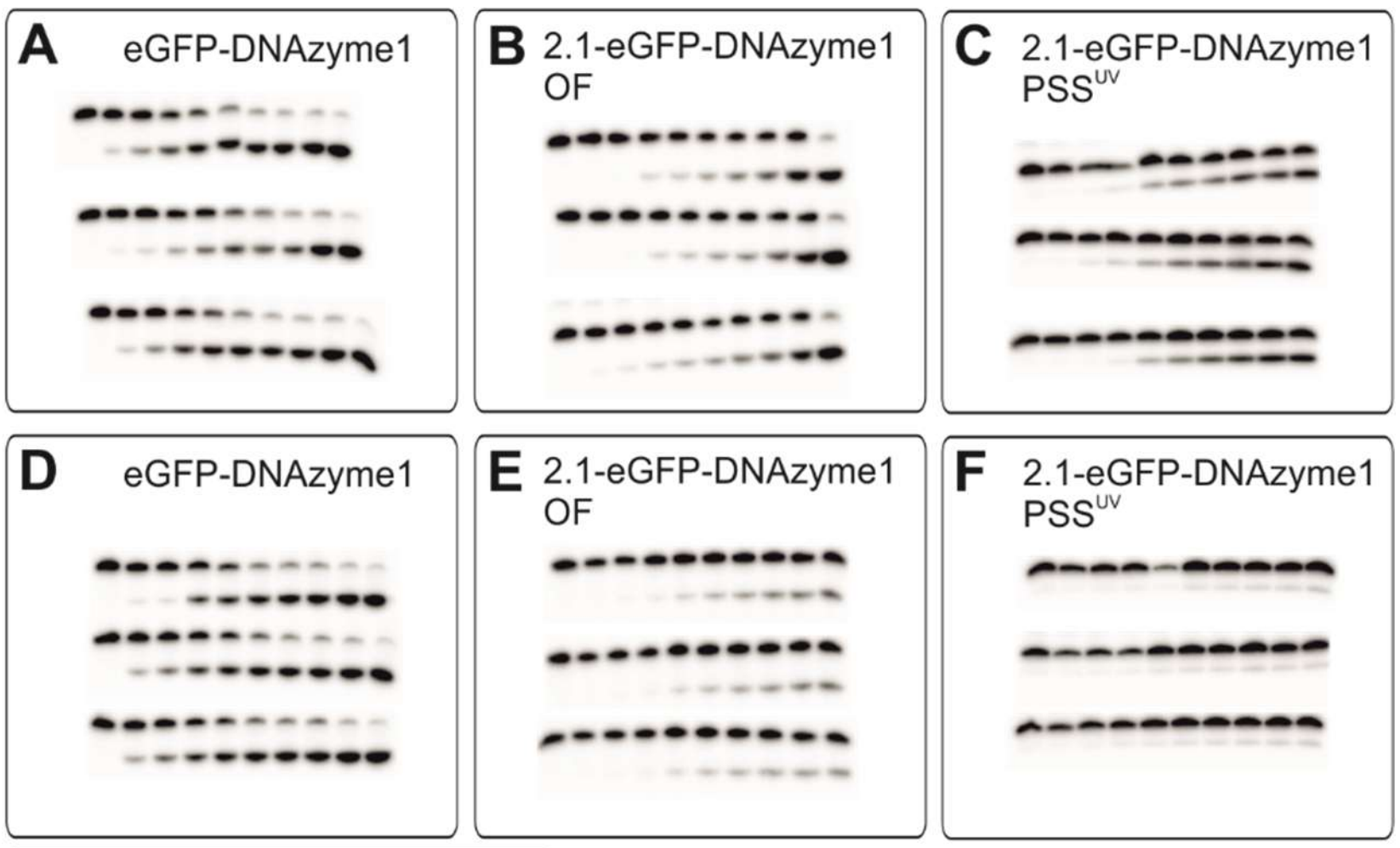

G

Modification	Mean v_0 [%/min]	Standard Deviation [%/min]
DNAzyme	26.45	2.344
2.1 OF	12.57	1.145
2.1 PSS	1.126	0.132
eGFP1	20.02	0.7375
eGFP1_OF	4.592	0.19326
eGFP1_PSS	0.37	0.01373
eGFP2	20.09	0.8778
eGFP2_OF	0.20274	0.0058
eGFP2_PSS	0.026	9.4E-4

Figure S13. Original data relating to Figure 5. A) Aliquots for the unmodified eGFP-DNAzyme1 as positive control. Time points are: 0, 0.5, 1, 2, 3, 5, 7, 10, 20, and 60 minutes. B) Aliquots for the 2.1-Ph^{t}Bu modified eGFP-DNAzyme1 in OF. Time points are: 0, 0.5, 1, 2, 3, 5, 7, 10, 20, and 60 minutes. C) Aliquots for the 2.1-Ph^{t}Bu modified eGFP-DNAzyme1 in PSS^{UV}. Time points are: 0, 2, 6, 12, 30, 50, 70, 90, 110, 130 minutes. D) Aliquots for the unmodified eGFP-DNAzyme2 as positive control. Time points are: 0, 0.5, 1, 2, 3, 5, 7, 10, 20, and 60 minutes. E) Aliquots for the 2.1-Ph^{t}Bu modified eGFP-DNAzyme2 in OF. Time points are0, 2, 6, 12, 30, 50, 70, 90, 110, 130 minutes. F) Aliquots for the 2.1-Ph^{t}Bu modified eGFP-DNAzyme1 in PSS^{UV}. Time points are: 0, 2, 6, 12, 30, 50, 70, 90, 110, 130 minutes. G) Exact values of plotted Data in Figure 5.

12. HPLC Analysis of the Closed Form

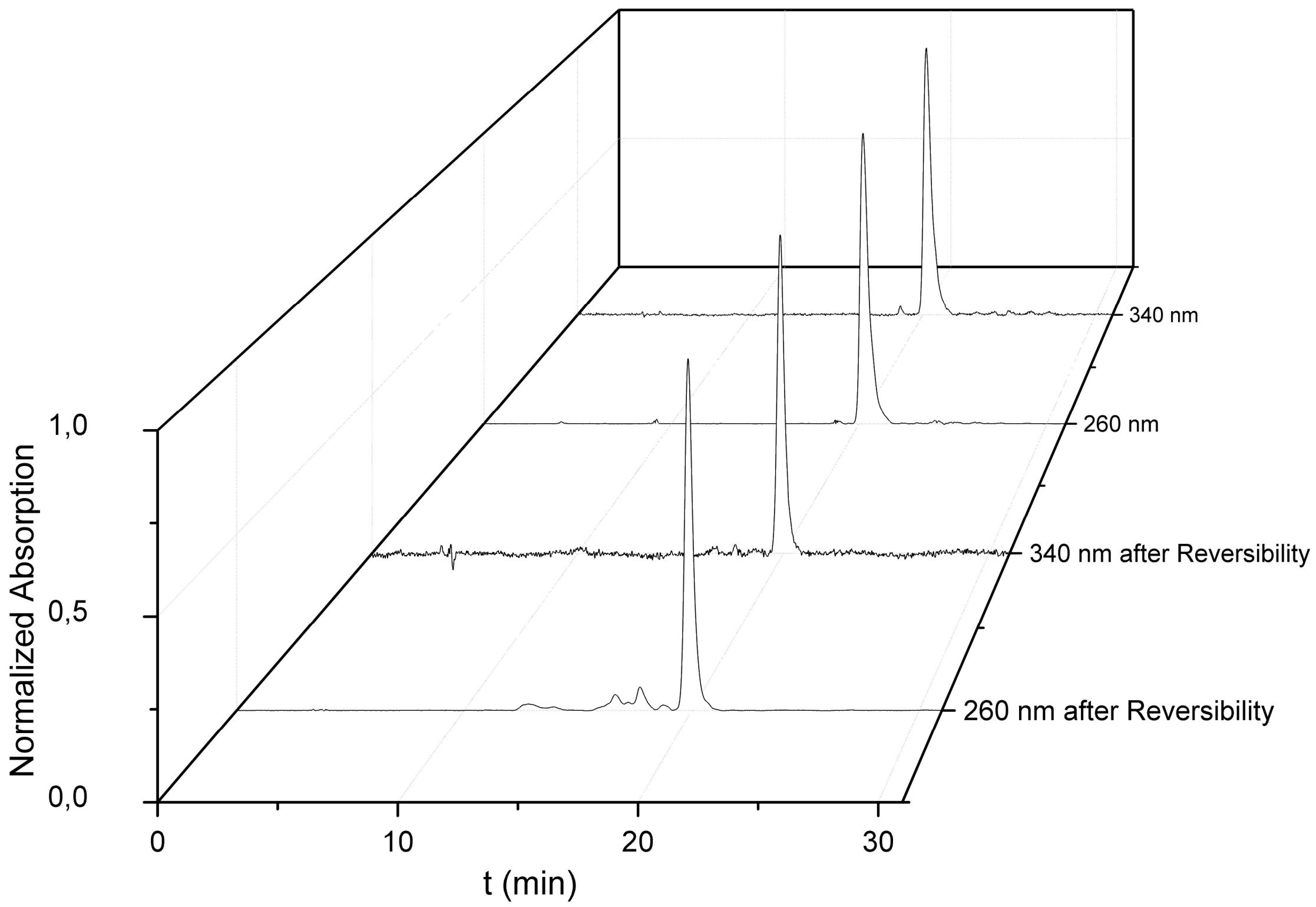

Figure S14. HPLC analysis of the 2.1-Ph^{t}Bu strand before and after the 40 cycle reversibility measurement. After 40 switching cycles, new peaks appear with absorption at 260 nm and/or 340 nm, indicating degradation.

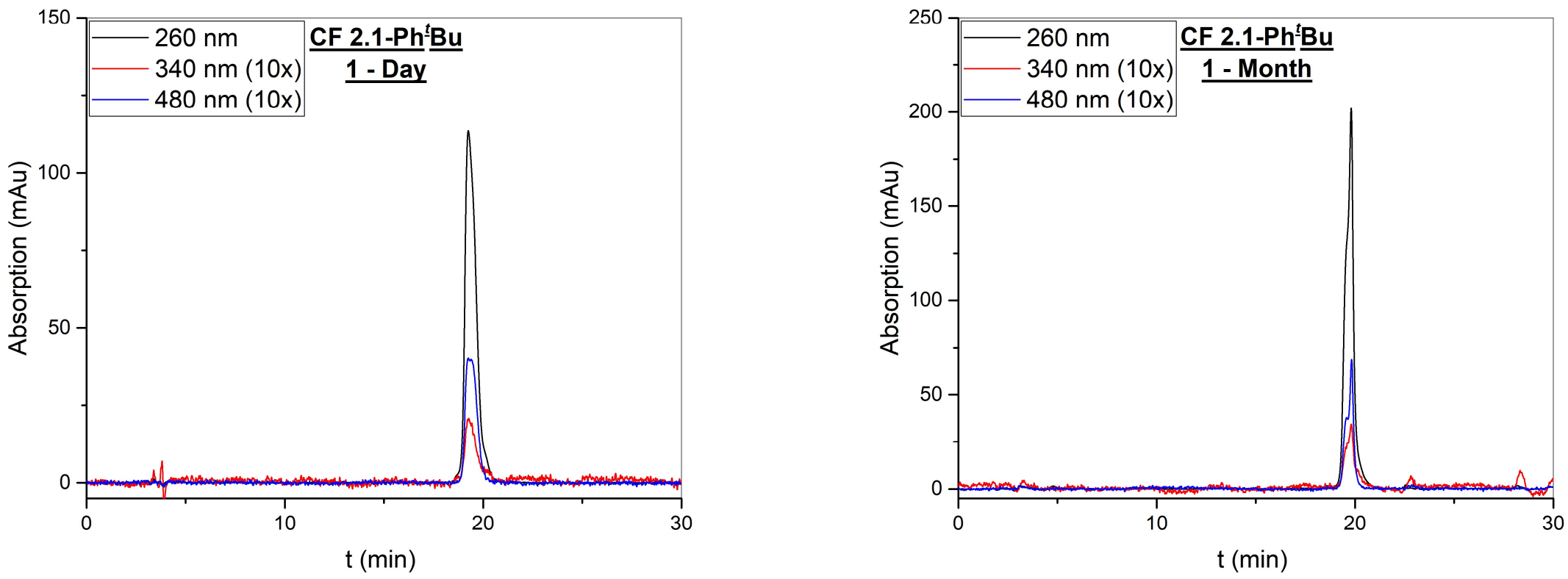

Figure S15. HPLC analysis of the isolated closed form which was stored at -21 °C wrapped in aluminum foil. HPLC analysis was performed after 1 day and 1 month of storage after isolation.

13. NMR Spectra

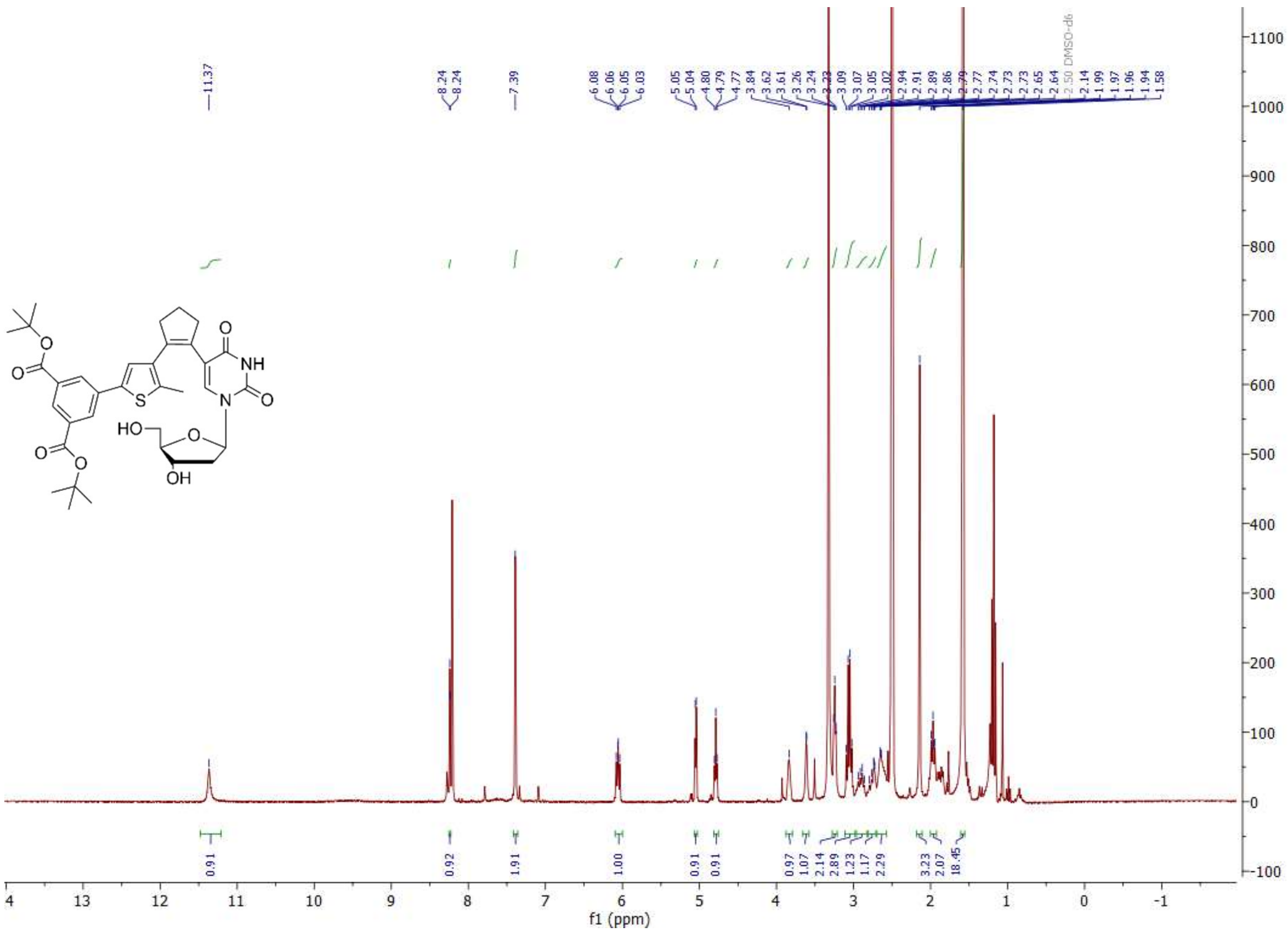

^{1}H NMR (300 MHz) of **6** in DMSO-d$_6$

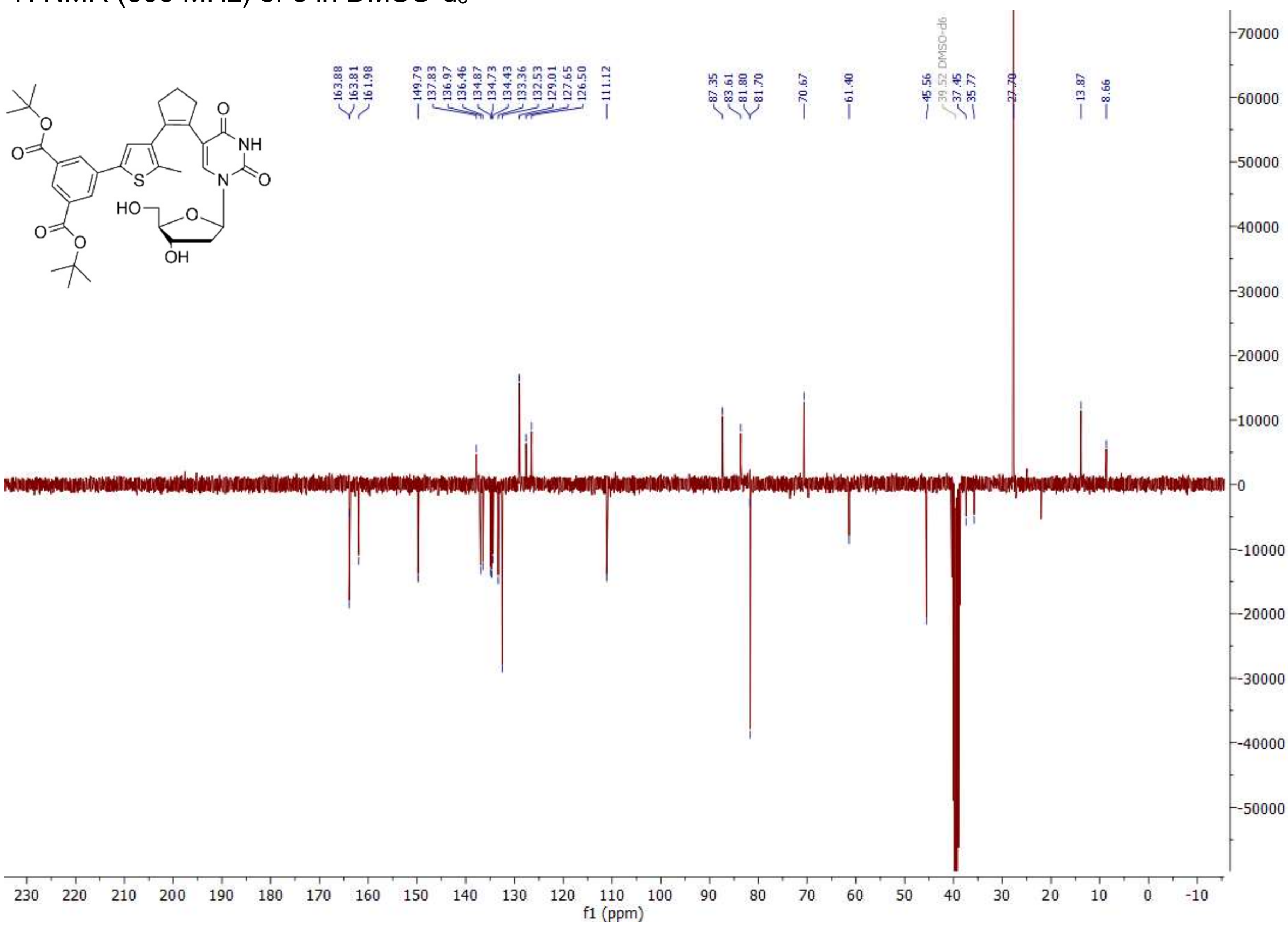

^{13}C NMR (126 MHz) of **6** in DMSO-d$_6$

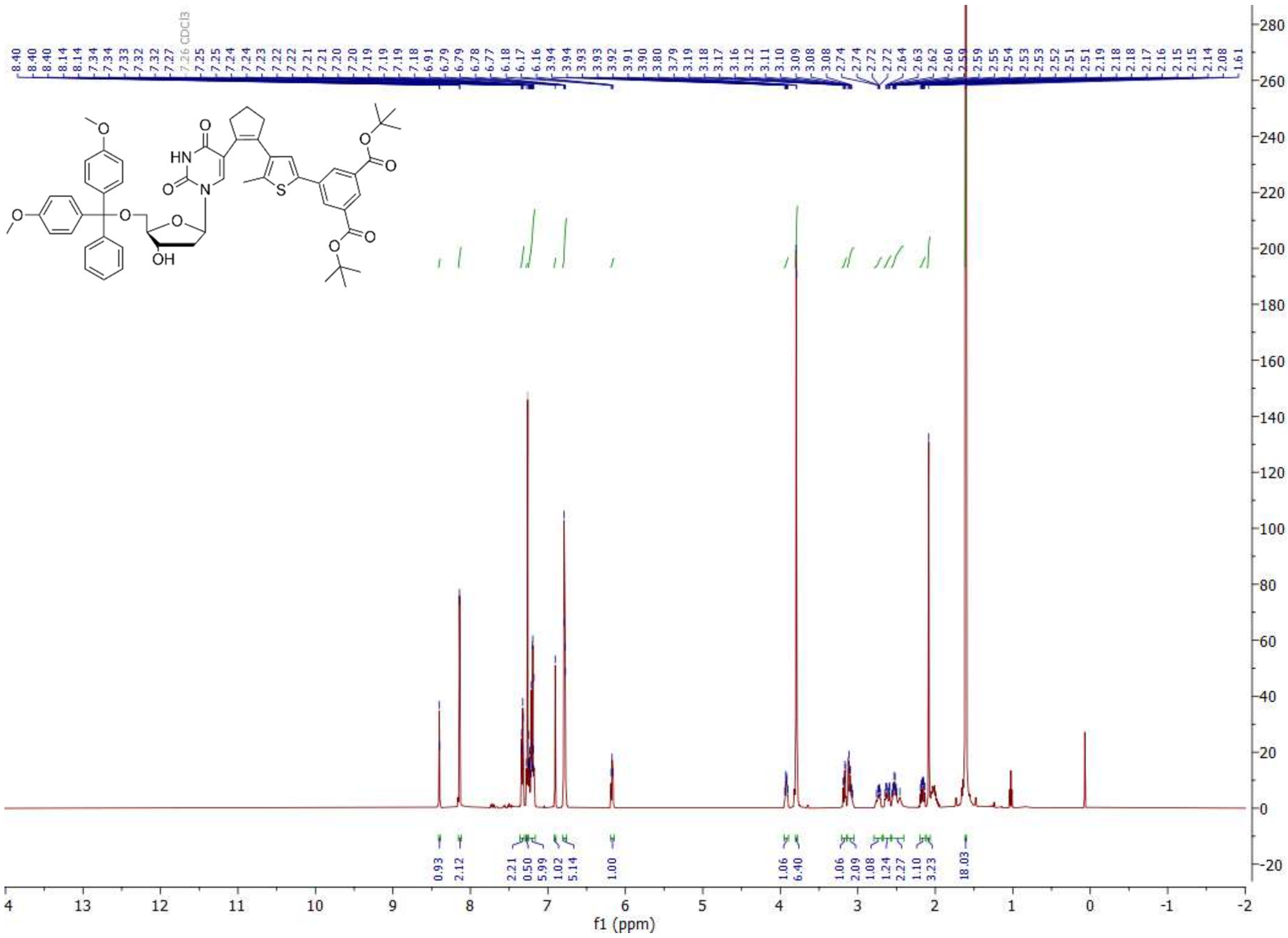

^{1}H NMR (500 MHz) of **8** in Chloroform-d

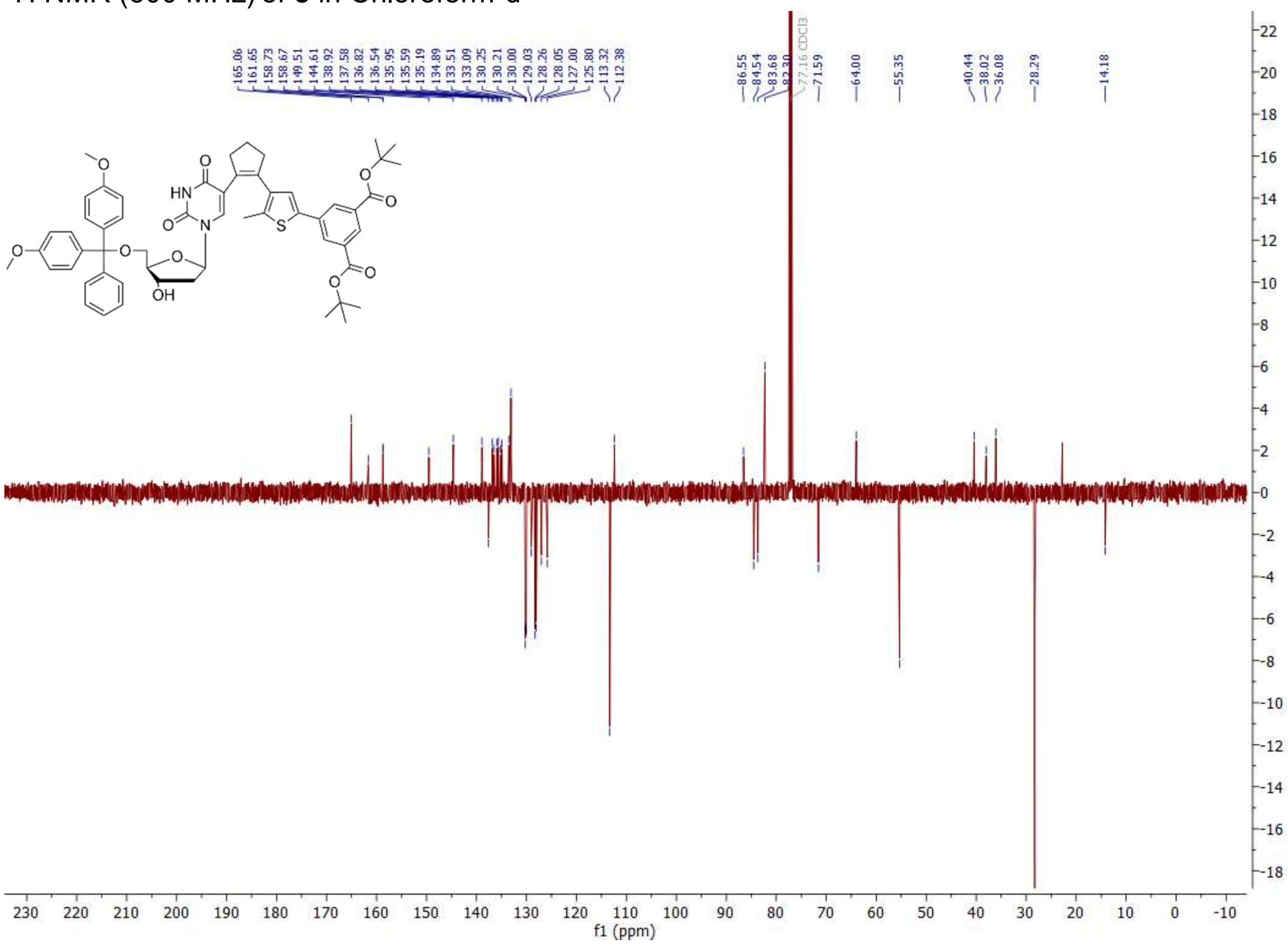

^{13}C NMR (126 MHz) of **8** in Chloroform-d

14. Supplementary References

[39] W. C. Still, M. Kahn, A. Mitra, "Rapid chromatographic technique for preparative separations with moderate resolution" *The Journal of Organic Chemistry* **43**, (1978): 2923-2925.

[40] U. Megerle, R. Lechner, B. König, E. Riedle, "Laboratory apparatus for the accurate, facile and rapid determination of visible light photoreaction quantum yields" *Photochemical & Photobiological Sciences* **9**, (2010): 1400-1406.

[41] C. M. Hendrich, M. Reinschmidt, S. M. Büllmann, T. Kolmar, A. Jäschke, "Synthesis and Development of Inverse-Type Nucleoside Diarylethene Photoswitches" *Chemistry–A European Journal* **30**, (2024): e202401537
